# Enhancing health-promoting isothiocyanates in Chinese kale sprouts via manipulating *BoESP*

**DOI:** 10.1093/hr/uhad029

**Published:** 2023-02-21

**Authors:** Huiying Miao, Chuchu Xia, Shunhao Yu, Jiansheng Wang, Yanting Zhao, Qiaomei Wang

**Affiliations:** Key Laboratory of Horticultural Plant Growth, Development and Quality Improvement, Department of Horticulture, Zhejiang University, Hangzhou 310058, China; Key Laboratory of Horticultural Plant Growth, Development and Quality Improvement, Department of Horticulture, Zhejiang University, Hangzhou 310058, China; Key Laboratory of Horticultural Plant Growth, Development and Quality Improvement, Department of Horticulture, Zhejiang University, Hangzhou 310058, China; Institute of Vegetables, Zhejiang Academy of Agricultural Sciences, Hangzhou 310021, China; Institute of Vegetables, Zhejiang Academy of Agricultural Sciences, Hangzhou 310021, China; Key Laboratory of Horticultural Plant Growth, Development and Quality Improvement, Department of Horticulture, Zhejiang University, Hangzhou 310058, China

## Abstract

Glucosinolates (GSLs) are a group of sulfur-containing secondary metabolites, which are abundant in *Brassica* vegetables. GSL breakdown products (GBPs), especially isothiocyanates (ITCs) benefit human health. Chinese kale is a native *Brassica* vegetable in China, and its sprouts are rich in GSLs and nutritional substances. ITCs are the predominant GBPs while alternative products are formed in the presence of specifier proteins. However, fewer ITCs are formed in the sprouts. Epithiospecifier (ESP) promotes the formation of epithionitriles at the expense of ITCs in *Arabidopsis*, but a systematic study of different isoforms of *ESP*s in most vegetables is still missing. In this study, changes in the content of GBPs and the precursor GSLs, as well as thiols per plant were monitored during sprout development. The proportions of epithionitriles and ITCs in total GBPs were found to be increased and decreased, respectively. RNA-seq showed enhanced expression of numerous genes involved in GSLs biosynthesis and degradation, as well as sulfur assimilation in sprouts compared to seeds. Four copies of *BoESP*s were isolated and *BoESP2* was the most abundant isoform. Generally, transcription of *BoESP*s showed a strong response to abscisic acid and gibberellin, and consequently epithionitriles increased under these treatments. Knockdown of *BoESP2* expression through virus-induced gene silencing system could effectively increase total ITCs and decrease total epithionitriles. Overall, dynamic GSL metabolic flux exists in the sprouting period, and the expression of *BoESP*s determines the pattern of GBPs, suggesting that improving the health-promoting ITCs in Chinese kale sprouts through manipulating *BoESP*s by metabolic engineering is feasible.

## Introduction

Sprout development starts from seed germination, which is an important stage in the plant life cycle. In this process, storage materials such as proteins, fats, and starches are degraded to small molecule compounds [1]. After this process, well developed sprouts are even more nutritious than the original seeds, which makes sprouts a popular vegetable [2]. Chinese kale (*Brassica oleracea var. alboglabra*) is a native Chinese *Brassica* vegetable, whose tender leaves and bolting stems are usually harvested for food. In recent years, Chinese kale sprouts are favored by producers and consumers because they are economical and rich in health-promoting compounds such as carotenoids, vitamin C, and glucosinolates (GSLs) which can be hydrolyzed by degradation enzymes to release breakdown products (GSL breakdown products, GBPs), conferring them additional health-promoting properties [3, 4]. Lots of work has been carried out to investigate the concentration of GSLs/GBPs at different plant developmental stages [5–10]. However, because the water content in sprouts at different developmental stage is different and is much higher than that in seeds, it is worth exploring the change of GSLs and GBPs per plant during sprout development to accurately report the metabolism of GSL and better access the health-promoting value of sprouts. To date, only a few studies have paid attention to the change in GSL content per plant during sprout development [11–13], and the change in GBP content per plant has not been reported.

GSLs are a group of plant secondary metabolites containing sulfur and nitrogen, which are mainly found in *Brassicaceae* plants, especially ecologically important *Brassica* crops. According to their amino acid precursors, GSLs can be classified into aliphatic GSLs (derived from methionine, alanine, leucine, isoleucine, and valine), indole GSLs (derived from tryptophan), and benzenic GSLs (derived from phenylalanine and tyrosine). Upon tissue damage, a group of beta-glucosidases termed myrosinases (thioglucoside glucohydrolases, TGGs) interact with GSLs and hydrolyze them into glucose and unstable aglucone [14]. The unstable aglucone undergoes spontaneous rearrangement and forms isothiocyanates (ITCs). Besides ITCs, thiocyanates, nitriles, and epithionitriles can be formed in the presence of thiocyanate-forming proteins (TFPs), nitrile specifier proteins (NSPs), and epithiospecifiers (ESPs). Among them, nitriles can be formed in the presence of NSPs, which is independent of the alkenylation of GSL structure, or in the absence of NSPs at low pH and high ferrous ion concentration [15–17]. ESP catalyzes alkenylated GSLs to epithionitriles and alkylated GSLs to nitriles [15, 18]. The well-known bioactivity of GSLs in plant defense and human health promotion is conferred by their hydrolysis products, particularly ITCs [19–22]. However, we found the GBPs in Chinese kale sprouts are mainly epithionitriles instead of much healthier ITCs in our previous study, indicating high ESP activity [4]. So far, the metabolic pathways of GSLs in model plant *Arabidopsis* have been fully studied [23]. However, in vegetables, research mainly focused on the biosynthesis of GSLs, such as clarifying the structural genes and regulatory factors (Miao, 2020), while the hydrolysis of GSLs has received limited attention. The function of *ESP* has been clarified in *Arabidopsis* since its first separation from *Crambe abyssinica* [24–27]. As *Brassica* species experienced a whole-genome triplication and very recent genome duplications after their divergence from *Arabidopsis* [28], the function of each *ESP* isoform might be more complex. In vegetables, so far partial *ESP* isoforms have been characterized in several *B. olearacea* cultivars, and their function has been analyzed through prokaryotic expression, or heterologous overexpression in *Arabidopsis* [18, 29–31]. Cloning and systematic study of all *ESP* isoforms in most vegetables are still missing.

In this study, we investigated the change of GBP pattern per plant from seed to 7-day-old seedling of Chinese kale to better evaluate the health-promoting value of Chinese kale sprouts. The underlying mechanisms were ascertained through studying GSL turnover and whole-genome transcriptome analyses. Also, the systematic characterization of all *ESP* isoforms in Chinese kale sprouts was carried out and in sprouts higher ITCs but lower epithionitriles were obtained via manipulating GBP pattern through virus-induced gene silencing (VIGS)-mediated gene silence of *BoESP*.

## Results

### Epithionitriles increase during sprout development

**Figure 1:**
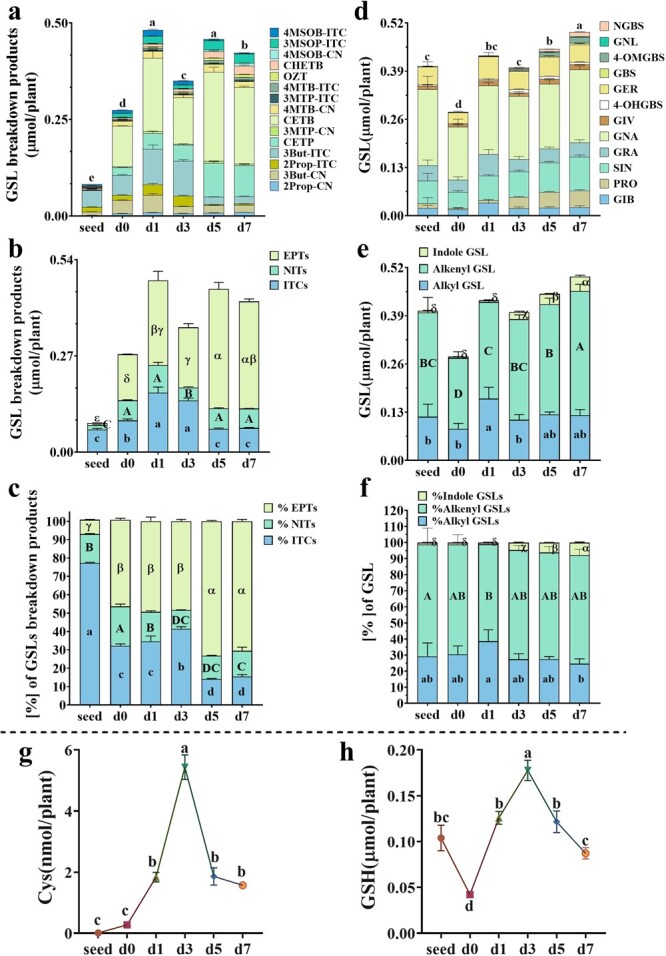
Changes of GSL breakdown products (**a**–**c**), glucosinolates (**d**–**f**), and primary thiols (**g**, **h**) during sprout development of Chinese kale. Values represent means ± SD of four replicates. Values not sharing a common letter are significantly different at *P* < 0.05. Cys: cysteine; GSH: glutathione. The full name of GSLs and GSL breakdown products can be found in Table S1 (see online supplementary material).

A total of 15 kinds of GBPs were detected in seeds and seedlings of Chinese kale (Fig. 1a; Table S2, see online supplementary material). There were seven kinds of ITCs, five kinds of nitriles, and three kinds of epithionitriles. Generally, the compositions of GBPs were consistent in seeds and different stages of seedlings, except for the lack of 4-(methylsulfinyl) butyl ITC in seeds. In seeds, 3-butenyl ITC was the predominant one (0.044 μmol/plant, accounting for 55.4% of total GBPs), followed by 2-propenyl ITC (0.013 μmol/plant, accounting for 16.0% of total GBPs). After germination, 1-cyano-3,4-epithiobutane became the richest GBP, ranging from 0.105 to 0.231 μmol/plant, accounting for 34.3% to 50.4%. The second abundant one was 3-butenyl ITC in soaked seeds (d0) and early seedlings (1-day-old to 3-day-old, d1 to d3), and 1-cyano-2, 3-epithiopropane in late seedlings (d5 to d7), respectively.

Overall, the content of individual and total GBPs varied during sprout development. The content of total GBP increased sharply by 247.9% (seed to d0) and 75.8% (d0 to d1) in very early seedling stage, and then stabilized at a high level until d7, except for a remarkable decrease of 27.4% showing up in d3 seedlings. The content of total epithionitriles and nitriles varied similarly to the total GBPs, while total ITCs went up first but then went down, with a peak at d1 (Fig. 1b). However, the proportion of ITCs in total GBPs dropped dramatically from seed (76.5%) to d0 (32.2%), and then showed a wavelike decrease during the rest days, accounting for 15.4% in d7 seedlings. In contrast, the proportion of epithionitriles increased along with seedling development, accounting for 71% in d7 seedlings (Fig. 1c).

### Dynamic changes in the content of glucosinolates and thiols during seedling development period

To explore why the content of epithionitriles kept rising and ITCs showed a declining trend during seedling development, we measured the content of precursor GSLs and thiols which correlate closely to GSLs. As shown in Fig. 1d and Table S3 (see online supplementary material), 12 GSLs were detected in seeds and all different ages of seedlings, including eight aliphatic GSLs and four indole GSLs. No benzoic GSL was found. During seedling development, aliphatic GSLs were always the predominant GSL, accounting for 92.1%~98.9% of total GSLs (Fig. 1f). Among them, the content of alkenyl GSL was 1.563 to 2.750 times higher than that of alkyl GSL (Fig. 1e), with gluconapin being the richest one (accounting for 39.0% to 51.0%), followed by sinigrin and glucoerucin (Fig. 1d).

From seed to d7, total GSL level in seeds plummeted to 0.134 μmol/plant after being soaked for one day, which was only 69.3% of that in seeds (Fig. 1d). It then maintained the upward trend overall, and finally reached 0.248 μmol/plant in d7 seedlings. The changes in the content of total aliphatic GSL and alkenyl GSL were similar to that of total GSLs, and alkyl GSL content changed little except for an increase at d1 (Fig. 1e). The content and proportion of indole GSL remained unchanged from seed to d1, and then kept rising (Fig. 1e and f).

The backbone of GSLs harbor two to three S atoms [32]. Primary sulfur metabolites, cysteine and glutathione, offer reduced sulfur in the biosynthesis of GSL. The hydrolysis of GSL, in turn, provides sulfur for the biosynthesis of primary sulfur metabolites [33, 34]. In this study, during seedling development, the content of cysteine went up first but then went down, with a peak at d3 (5.4 nmol/plant) (Fig. 1g). From seed to d7, the change of glutathione content was wavelike. The lowest level was observed in d0 seedlings (0.04 μmol/plant), while the highest one was in d3 seedlings (0.18 μmol/plant) (Fig. 1h). Taken together, these results indicated that dynamic sulfur metabolic flux exists during seedling development.

### Transcriptome analysis of GSL metabolism-related genes in Chinese kale seeds and seedlings

To further investigate the potential mechanism of GSL turnover, we conduct RNA-seq in seeds and d3 seedlings, as the accumulation of both cysteine and glutathione was highest in d3 seedlings, and there was a noticeable difference in GBP pattern between seeds and d3 seedlings (Fig. 1). A total of 20 667 differentially expressed genes (DEGs) were found, including 12 508 up-regulated and 8159 down-regulated genes (Fig. S2a, see online supplementary material). Kyoto Encyclopedia of Genes and Genomes (KEGG) pathway enrichment analysis revealed that many genes were enriched in signaling pathways closely to photosynthesis and energy metabolism, while ‘cysteine and methionine metabolism’ and ‘sulfur metabolism’ were also included in the top 20 of KEGG pathways (Fig. S2b, see online supplementary material).

**Figure 2:**
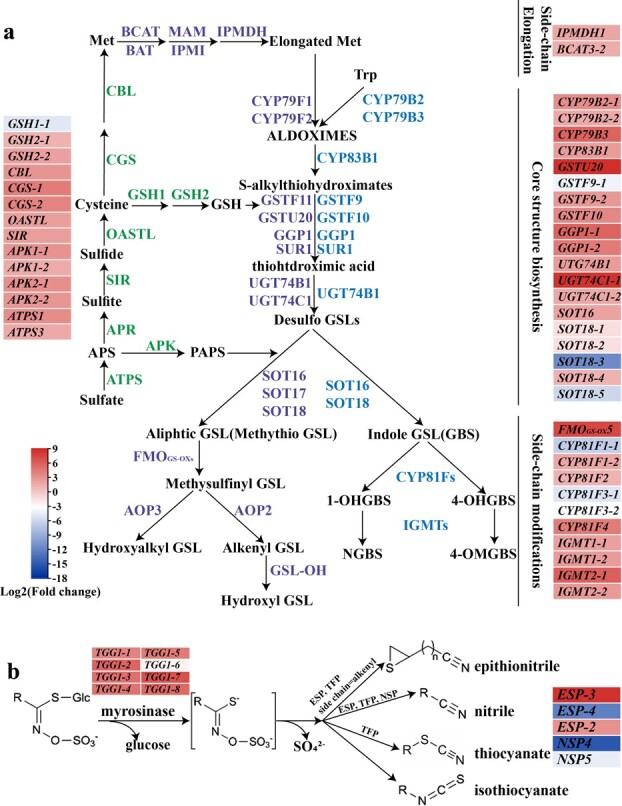
Changes of differentially expressed genes related to GSL synthesis and primary sulfur assimilation (**a**), as well as GSL breakdown pathway (**b**) in d3 vs seed. Values represent the means of three replicates. The expression levels were visualized by TBtools.

The DEGs involved in GSL turnover were paid close attention, namely GSL biosynthesis and degradation, as well as the sulfur assimilation pathway. Overall, a total of 32 DEGs related to GSL biosynthesis were identified, and most of them showed higher expression level in d3 seedlings than in seeds (Fig. 2a). Among them, two DEGs (*IPMDH1* and *BCAT*3) were involved in side-chain elongation of aliphatic GSL, and 19 DEGs involved in the core structure biosynthesis of GSL, including nine DEGs participated in both aliphatic and indole GSL biosynthesis, and three and seven DEGs were linked to aliphatic GSL and indole GSL, respectively*.* In addition, 11 DEGs were involved in the side-chain modification of GSL, including aliphatic GSL-related *FMO_GS-OX_*, indole GSL-related *CYP81F* and *IGMT*. For sulfur assimilation pathway, we identified a total of 14 DEGs that almost covered the whole pathway, including *ATPS*, *APK*, *SIR*, *OASTL*, *CGS*, *CBL*, *GSH1*, and *GSH2*. All of them had higher expression level in d3 seedlings than in seeds, except for *GSH1–1* (Fig. 2a).

For classical GSL degradation pathway, a total of 13 DEGs were identified, including eight *TGG1*s, three *ESP*s, and two *NSP*s (Fig. 2b). Almost all identified *TGG1*s showed higher expression levels in d3 seedlings than in seeds, which might facilitate more myrosinases accumulation in d3 seedlings than in seeds. ESP and NSP have been reported to promote the formation of epithionitriles and nitriles [35]. Here, only the expression of *ESP* was observed higher in d3 seedlings than in seeds, indicating that *ESP* might be the key to explaining higher content of epithionitriles and nitriles in d3 seedlings than that in seeds. Taken together, these results confirmed our findings of the dynamic metabolic flux at the transcriptional level, and *ESP* might be the vital gene affecting the pattern of GBPs in Chinese kale sprouts.

### Four copies of *BoESP* were isolated from Chinese kale

The putative homologous *ESP* protein sequences from cabbage, namely *BoESP1*^cabbage^ (Bol039072), *BoESP2*^cabbage^ (Bol006380), *BoESP3*^cabbage^ (Bol024137) and *BoESP4*^cabbage^ (Bol013374), were identified through the BLASTP program in BRAD by using *Arabidopsis ESP* amino acid sequence as a query sequence. Then, four copies of *ESP*, being named *BoESP1*, *BoESP2*, *BoESP3*, and *BoESP4*, were isolated from Chinese kale by referring to the four *ESP* homologous sequences of cabbage. The coding sequences of *BoESP1*, *BoESP2*, and *BoESP3* were 1029 bp, and *BoESP4* was 1044 bp (Fig. S3, see online supplementary material). The multiple sequence alignment analysis showed that the protein sequences of the four *BoESP*s were 100% identical with the corresponding *ESP*s from cabbage, and more than 75% identical with *AtESP* (Fig. S4, see online supplementary material). Even the identity between every two *BoESP*s was more than 80%. The phylogenetic tree was constructed based on *ESP*s from *Arabidopsis*, Chinese kale, and six species in U’s triangle (Fig. S5, see online supplementary material). *BoESP1*, *BoESP2*, and *BoESP3* were grouped into a sub-group, which was separated from *BoESP4*. Generally, *BoESP*s were grouped most closely with the *ESP*s from *B. oleracea*, *Brassica napus*, and *Brassica carinata*, rather than *Brassica nigra*, *Brassica rapa*, and *Brassica juncea*.

### Subcellular localization and spatio-temporal expression pattern of *BoESP*s

**Figure 3:**
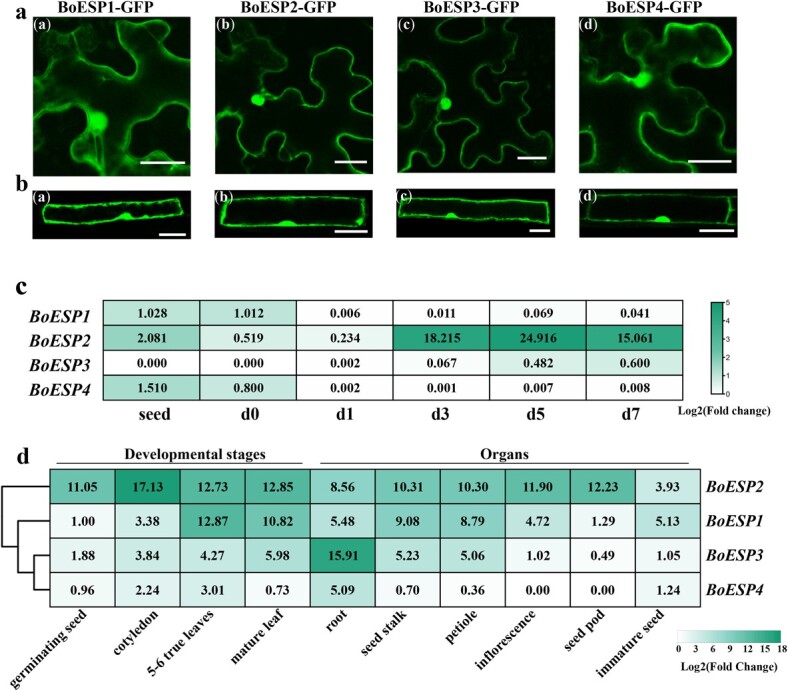
Subcellular localization analysis of *BoESP* in tobacco leaves (**a**) and *Arabidopsis* root cells (**b**). Changes of *BoESPs* expression during sprout development (**c**). Expression pattern of *BoESP* in various developmental stages and organs of mature plants (**d**). Scale bars represent 25 μm. The relative expression level of *BoESP1* in seed (**c**) or germinating seed (**d**) was set as 1. Values represent means of three replicates.

To investigate the possible cellular location that individual *BoESP* function in, we carried out transient expression in tobacco leaf cells (Fig. 3a). Generally, when *BoESP*s were expressed in GFP fusions under the control of the *Cauliflower mosaic virus* (CaMV) 35S promoter, all proteins distributed throughout the cytoplasm and the nucleus. These results were confirmed by heterologous overexpression of BoESP-GFP in *Arabidopsis* (Fig. 3b). The expression of *BoESP2* was the highest in almost all stages of seedling development, and it was much higher in d3 seedlings (approximately 8.75-fold) than in seeds, which was in accordance with the results of RNA-seq. The expression of *BoESP1*, *BoESP3*, and *BoESP4* was quite low during sprout development (Fig. 3c). The expression of all four *BoESP*s in different developmental stages of young plant and different organs of mature plants showed that *BoESP2* was the isoform with the highest expression, followed by *BoESP1*, except that *BoESP3* was strongly expressed in the root of the mature plant. Besides, the overall expression of *BoESP4* was extremely weak and was undetectable in inflorescence and seed pod (Fig. 3d).

### The expression of *BoESP*s and the pattern of GBPs in response to plant hormones

**Figure 4:**
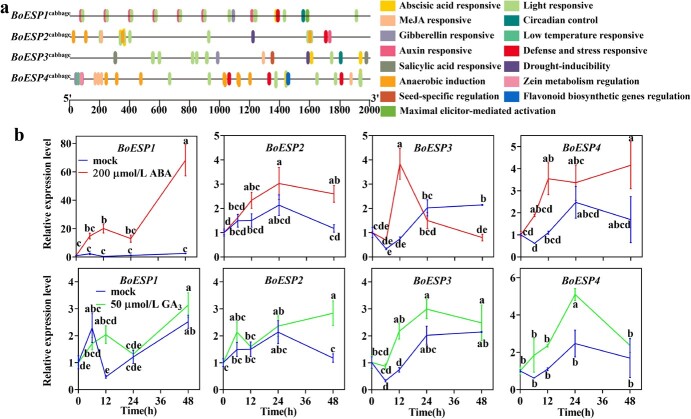
*Cis*-element analysis of the *ESP* promoters from cabbage (**a**). Effect of abscisic acid and gibberellin on *BoESP*s expression in Chinese kale sprouts (**b**). The relative expression level of *BoESPs* in mock-treated sprouts was set as 1. Values represent means ± SD of three replicates. Values not sharing a common letter are significantly different at *P* < 0.05.

The analysis of *cis*-acting elements in the promoter region of *ESP*s was carried out to better understand the regulatory mechanisms involved in *ESP* expression. As the coding sequences of *ESP*s exhibit extreme conservation between Chinese kale and *B. oleracea*, the promoter sequences of *B. oleracea* from BRAD were employed to conduct the analysis. As shown in Fig. 4a and Table S4 (see online supplementary material), abundant hormone-responsive *cis*-elements were identified in the promoter region of all four *ESP*s from cabbage with a difference in quantity and variety. Specifically, there existed abscisic acid (ABA) and methyl jasmonate (MeJA)-responsive *cis*-elements in the promoter region of *BoESP2*^cabbage^, *BoESP3*^cabbage^ and *BoESP4*^cabbage^; gibberellin (GA)-responsive *cis*-elements in *BoESP1*^cabbage^, *BoESP3*^cabbage^ and *BoESP4*^cabbage^; auxin-responsive *cis*-elements in *BoESP1*^cabbage^, *BoESP2*^cabbage^, and *BoESP4*^cabbage^; salicylic acid (SA)-responsive *cis*-elements in *BoESP3*^cabbage^*.* It is noteworthy that the promoter region of all four genes contained many light-responsive *cis*-elements.

**Figure 5:**
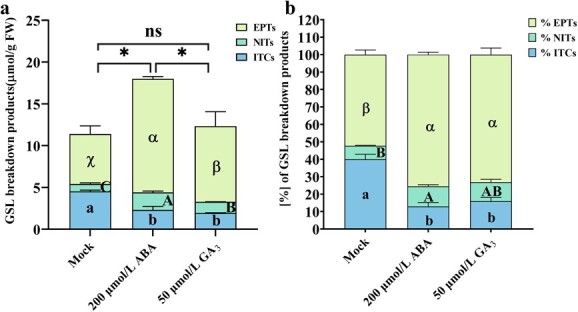
The content (**a**) and proportion (**b**) of GSL breakdown products in response to abscisic acid and gibberellin treatments in Chinese kale sprouts. Values represent means ± SD of three replicates. ^*^ and values not sharing a common letter are significantly different at *P* < 0.05.

We then experimented to verify the response of *BoESP*s to plant hormones by exogenous application of ABA, GA_3_, and SA on Chinese kale sprouts, as they represent the richest *cis*-element in the promoter of *BoESP2*^cabbage^, the *cis*-element that exists in the promoter of other three *ESP*s from cabbage, and the one that only exists in the promoter of *BoESP3*^cabbage^. As shown in Fig. 4b, the expression of four *BoESP*s homologs could be induced by ABA treatment, with *BoESP1* being the strongest one. The expression of *BoESP1* was increased significantly by 41.519- and 26.020-fold, respectively, at 12 h and 48 h after ABA treatment. The transcription level of *BoESP2* was significantly increased after 48 h treatment of ABA, which was 2.211 times higher compared to the control. The expression of *BoESP3* responded positively to ABA treatment at the early stage (before 12 h). Compared to mock, *BoESP4* showed a higher expression level upon ABA treatment, but it is not statistical significance. For GA_3_ treatment, the expression of *BoESP*s could be induced generally. The transcription levels of *BoESP2*, *BoESP3*, and *BoESP4* were significantly enhanced by 14.17%, 19.22%, and 10.64%, respectively, at 48 h, 12 h, and 24 h after GA_3_ treatment. However, no significant induction of SA on *BoESP*s expression was observed in this study (Fig. S6, see online supplementary material). Taken together, these results indicate the expression of *BoESP*s can be effectively induced by 200 μmol/L ABA and 50 μmol/L GA_3_.

Next, we measured the changes in GBPs in response to ABA and GA_3_ treatment. Both hormone treatments did not affect the GBPs profiles but changed the content of individual GBPs considerably in Chinese kale sprouts (Table S5, see online supplementary material). After ABA and GA_3_ treatment, the predominant 1-cyano-3,4-epithiobutane was remarkably increased by 1.554- and 0.414-fold, respectively, while the accumulation of total epithionitriles was also notably accelerated by 1.271- and 0.513-fold; the content of total nitriles was significantly boosted by 1.346- and 0.497-fold, respectively (Fig. 5a, see online supplementarymaterialTable S5). However, the content of total ITCs was decreased markedly by 48.72% and 56.88%, respectively (Fig. 5a). In general, ABA treatment promoted the formation of total GBPs while GA_3_ did not. We also calculated the proportion of each kind of GBPs in total GBPs. Upon ABA and GA_3_ treatment, the proportion of epithionitriles was increased by 44.44% and 39.86%, respectively, while ITCs was decreased by 67.67% and 59.97%, respectively (Fig. 5b). The proportion of nitriles was increased significantly upon ABA treatment. Overall, our findings suggest ABA and GA_3_ regulate the pattern of GBP by affecting the expression level of *BoESP*s. 

**Figure 6:**
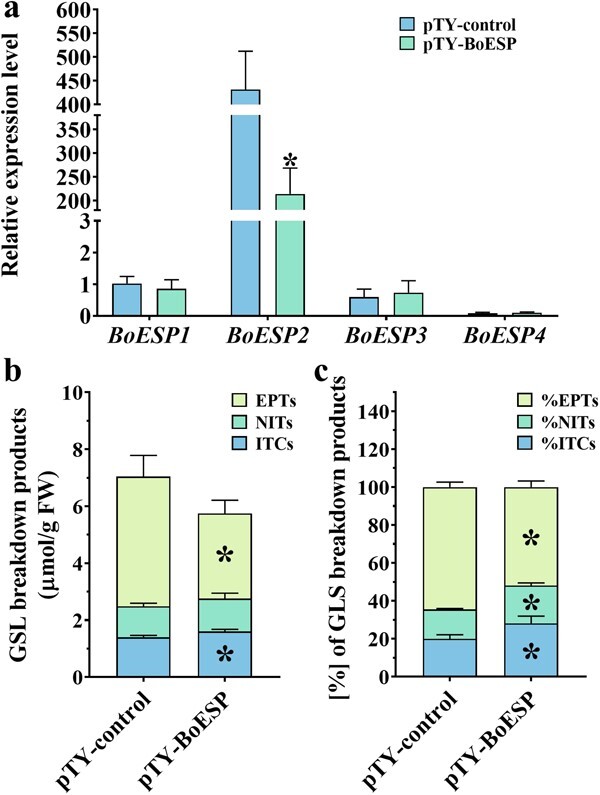
The expression level of *BoESP* in the control group (pTY-control) and *BoESP*-silenced plants (pTY-BoESP) (**a**); Epithionitriles, nitriles and isothiocyanates ratio in the control group and *BoESP*-silenced plants (**b**). The relative expression level of *BoESP1* in control group was set as 1.Values represent means ± SD (*n* = 3–9). Values with ^*^ are significantly different at *P* < 0.05.

### VIGS-mediated gene silencing of *BoESP* enhances the accumulation of ITCs and reduces the content of epithionitriles

To ascertain the function of *ESP* in GBPs formation in Chinese kale sprouts, we employed the VIGS system to generate Chinese kale sprouts with decreased expression of *BoESP*s. A batch of material with decreased expression of *BoESP*s was obtained, and those with *BoESP2* being decreased by approximately 50% were selected for further detection (Fig. 6a). GBPs detection showed that *BoESP2*-silenced sprouts had comparable content of total GBPs to the control (Fig. 6b). Specifically, the contents of total ITCs were significantly increased by 15.03%, and total epithionitriles were decreased by 34.37% in *BoESP2*-silenced sprouts, while no significant change was observed in total nitriles. From the perspective of proportion, total epithionitriles in *BoESP2*-silenced sprouts consumed a smaller proportion in total GBPs when compared to the control, whereas the proportion of both total nitriles and ITCs was higher (Fig. 6b). Thus, these results indicate that *BoESP2* plays an important role in the formation of epithionitriles, but not nitriles.

## Discussion

In this study, we measured the content and composition of GBPs throughout sprout development and calculated the level per plant. Generally, sprouts form more ITCs and total GBPs than seeds (Fig. 1a and b). Considering the constantly rising biomass of sprouts, d1 sprouts are recommended to harvest for providing ITCs or total GBPs as fewer of them can generate more GBPs. To date, extremely few studies have been devoted to monitoring the changes of GBPs during the sprouting period and a unified conclusion has not been formed yet. In broccoli, Williams *et al.* (2008) reported a general decrease in sulforaphane and sulforaphane nitrile concentration (μmol/g FW) during the first 7 days of seedling development, while Gu *et al.* (2012) observed that the concentration of sulforaphane (mg/g DW) went down sharply during the first day of seed germination, then went up and reached the same level as that in seeds at 48 h before decreasing again [36, 37]. In the current survey, the content of total ITCs per plant showed an inverted V-shaped trend throughout the whole Chinese kale sprouting period. It might be the different representation methods that resulted in inconsistent conclusions among different studies. To our surprise, more epithionitriles and nitriles were observed in sprouts than in seeds, and both the content and proportion of epithionitriles exhibited an increasing trend. This is not beneficial to Chinese kale sprout quality because ITCs have stronger potential health-promoting effects [19, 20, 22]. In contrast to GBPs, the content of precursor substances GSL dropped sharply from seeds to d0 seedlings (Fig. 1d and e). This is in agreement with the observations in several *Brassica* crops [6]. Correspondingly, only a trace level of cysteine was detected and the content of glutathione dropped sharply. It might be because GSLs and glutathione were hydrolyzed to provide substrates for the synthesis of life’s basic chemicals to ensure survival and growth because sprouts cannot harvest energy and chemicals from photosynthesis at this stage. As it has been reported, GSLs are an important source of sulfur, which can be degraded by β-glucosidase BGLU28 and BGLU30 to provide sulfur for cysteine synthesis [33]. Low level of cysteine might be due to active sulfur metabolic flux that cysteine turns into other urgent chemicals quickly as it is the precursor of massive biomolecules and possesses low-accumulation but high-flux property [38, 39]. However, the reason why high GSLs generate low GBPs in seeds is probably because the hydrolysis product of GSLs may be further converted to other chemicals in the seed homogenate [40]. Besides, from d0 to d7, the content of total GSLs changed similarly to that of total GBPs, but the proportion of alkenyl GSLs and alkyl GSLs was kept almost unchanged while that of ITCs decreased and epithionitriles increased. Correspondingly, the transcription of *ESP*s rose to a higher level in the later stage of sprout development (Figs 2b and 3c). Thus, it might be the enhancement of *ESP* that led to more epithionitrile formation. Moreover, during seed germination and seedling growth, radicle protrudes through the seed coat and the interaction between plants and environments increases gradually. Therefore, more defense chemicals like GSLs are needed to resist biotic and abiotic stresses. Following this, we found the content of total GSLs kept rising from d0 to d7 (Fig. 1d and e). However, conflicting results have been observed in *Arabidopsis*, which were planted in the nutritional matrix [11], suggesting that circumstance conditions affect the GSL metabolism greatly. Interestingly, the content and proportion of indole GSL increased markedly from d3 to the end of the experiment (Fig. 1e and f). Consistently, RNA-seq results showed that more GSL-related up-regulated DEGs are indole GSL biosynthetic genes. As the young seedling has been well developed 3 days after sowing, more biotic stresses will be encountered, thus, more defense substances are needed, particularly indole GSL which is important in the resistance to herbivorous insects and pathogens. Hanschen *et al.* (2017) compared the content of GSLs between sprouts and fully developed heads of five kinds of *Brassica* crops, and found that the latter had higher indole GSL content [41]. What’s more, Wiesner *et al.* (2013) demonstrated that mature leaves accumulated more indole GSL than sprouts in Pak Choi [42]. All these reports suggest that plants increase indole GSL storage along with growth and maturity.

We identified and characterized four *BoESP* isoforms in Chinese kale. High identity between *BoESP*s and *AtESP* was observed (Fig. S4, see online supplementary material), and the amino acid sequences of *ESP* from oilseed rape and broccoli were more than 75.0% identical with *AtESP* [18, 29]. These results suggest that *ESP* is highly conserved in the *Brassicaceae* family. Both transient expression in tobacco leaf cells and heterologous overexpression in *Arabidopsis* showed clear cytoplasmic and nuclear localization of *BoESP*, which is in line with the localization of *AtESP* in *Arabidopsis* [24]. This cytoplasmic and nuclear localization facilitates the dual functions of *ESP* as an epithiospecifier involved in GSL degradation and regulator of WRKY53 involved in leaf senescence [43]. Spatio-temporal expression pattern of *BoESP*s in Chinese kale indicated that *BoESP3* was rich only in root, while *BoESP1* and *BoESP2* were abundant in other tested tissues. A similar result has been found by Witzel *et al.* (2019) in white cabbage [31]. However, *AtESP* was detected in all organs of *Arabidopsis* (Ler) except for the roots [24]. It is known that the At-α whole-genome duplication occurred near the origin of the *Brassicaceae* family. Subsequently, retained gene duplicates of ancestral flavin monooxygenase glucosinolate S-oxygenase (FMO-GSOX) enzymes undertook tandem duplication and subfunctionalization to catalyze side chain modifications of Met-derived GSLs [44, 45]. There exists a monophyletic origin of ESPs from NSPs, and the split between them occurred as the consequence of the α-whole genome duplication followed by neofunctionalization [46]. Correspondingly, the generation of non-isothiocyanate GBPs is widespread in the *Brassicaceae*, but not common in plants of other families of the *Brassicales* [46]. Although isothiocyanates are more toxic than their corresponding nitriles, some specialist herbivores use isothiocyanates as cues to identify host, thus specifier proteins may confer additional resistance to specialist herbivores on plants [21, 46]. As *Brassica* species experienced genome duplications after their divergence from *Arabidopsis* [28], different isoforms of *ESP*s in *Brassica* vegetables may evolve diverse functions in specific tissues to better adapt to the environment or selection pressures from specialist herbivores. Overall, the expression of *BoESP*s in various organs throughout the whole plant implies an indispensable role in the life cycle.

Exogenous ABA and GA_3_ treatments caused notable *BoESP*s induction. Correspondingly, the content and proportion of epithionitriles were significantly increased by ABA and GA_3_ treatments, while those of ITCs were decreased markedly. Besides, the content of total GBPs was boosted by ABA application but not GA_3_, which is consistent with previous reports that ABA treatment rather than GA_3_ enhanced the accumulation of GSL [47, 48]. Thus, increasing the content and proportion of ITCs in Chinese kale sprouts through inhibition of GA_3_ signaling might have potential to improve the health-promoting quality of Chinese kale sprouts. Overall, these results imply that the function of *ESP* in promoting the formation of epithionitriles at the expense of ITCs might be conserved in Chinese kale [18, 26]. This was testified *in vivo* as *BoESP2*-silenced sprouts showed a significant decline of epithionitriles, and an increase of ITCs in comparison to the control (Fig. 6). Besides, the content of nitriles did not change significantly when the expression of *BoESP2* was knocked down, suggesting *BoESP2* is mainly in charge of the formation of epithionitriles rather than nitriles in Chinese kale sprouts.

In summary, dynamic GSL metabolic flux exists during sprout development to fine-tune the growth and resistance. The content and proportion of total epithionitriles increased along with sprout development, while ITCs showed a decreasing trend. However, this pattern could be altered by regulating *BoESP*s via VIGS-mediated gene silence, implying the possibility of artificially improving the health-promoting quality of Chinese kale sprouts.

## Materials and methods

### Plant materials, growth conditions, and chemical treatments

The seeds of Chinese kale (*B. oleracea var. alboglabra* Bailey cv. Sijicutiao) were sterilized in 20% bleach for 10 min, and washed with distilled water 5–6 times. Then they were immersed in distilled water for 24 hours at 25°C without light. Sprouts were grown in plastic trays with distilled water at 23°C under a 16 h/8 h light/dark cycle with 80% relative humidity (Fig. S1, see online supplementary material). Distilled water in the plastic trays was replaced every third day, and no fertilizer was added. Sprouts were harvested 0 day (d0), 1 day (d1), 3 days (d3), 5 days (d5), and 7 days (d7) after sowing. The dry seeds and harvested sprouts were frozen in liquid nitrogen immediately. Part of them was lyophilized by a freeze-dryer and was crushed into powder for GSL analysis, while the rest were stored at −80°C for the detection of GBP and thiols. Besides, the weights of 80–300 fresh or dried plants at each time point were recorded for the calculation of the weight per plant.

Chinese kale for spatio-temporal expression detection were grown in soil, and samples at different developmental stages and organs were harvested, frozen in liquid nitrogen immediately, and then stored at −80°C for RNA extraction.

For chemical treatments, Chinese kale seeds were sterilized and soaked as above. Sprouts were grown in plastic tissue culture vessels (7 × 8 × 7 cm) with five pieces of wet absorbent gauze in the same chamber as Chinese kale sprouts. Four days after sowing, sprouts were watered with 4 mL 200 μmol/L abscisic acid or 4 mL 50 μmol/L or sterile-distilled water (control group). Sprouts were harvested at 0 h, 6 h, 12 h, 24 h, and 48 h after treatment for RNA extraction, and 72 h after treatment for GBP detection.

### Gene cloning and sequence analysis

Primer pairs of BoESP1F-BoESP1R, BoESP2F-BoESP2R, BoESP3F-BoESP3R, and BoESP4F-BoESP4R (Table S6, see online supplementary material), respectively, were used for *BoESP*s amplication, which were designed according to the sequences of *ESP*s from cabbage obtained from the Brassicaceae Database (BRAD) (http://brassicadb.cn). The cDNA of Chinese kale sprouts was used as the template, and PrimeSTAR® HS DNA Polymerase (premix) (Takara, Japan) was used for DNA amplification. The amino acid sequences of *ESP* from other species were acquired from BRAD and The Arabidopsis Information Resource (TAIR). Multiple sequence alignment of *BoESP*s, *ESP*s from cabbage, and *AtESP*s was conducted by ClustalW (https://www.genome.jp/tools-bin/clustalw). The image showing the aligned sequences was generated by ENDscript/ESPript (https://espript.ibcp.fr/ESPript/cgi-bin/ESPript.cgi). MEGA 7.0 was employed to construct the phylogenetic tree with the neighbor-joining (NJ) method and bootstrap analysis (500 replicates). The results were expressed as a figure drawn by using iTOL (https://itol.embl.de/itol.cgi). The regulatory elements in the promoters were analysed by PlantCARE (http://bioinformatics.psb.ugent.be/webtools/plantcare/html/), and TBtools was used to visualize the results [49].

### Recombinant DNA techniques and plasmid construction

To produce BoESP1-GFP expression in tobacco leaves and Col-0 plants, primer pairs of aatB1-BoESP1-F and aatB2-BoESP1-R, aatB1-BoESP2-F and aatB2-BoESP2-R, aatB1-BoESP3-F and aatB2-BoESP3-R, aatB1-BoESP4-F and aatB2-BoESP4-R, respectively, were used to amplify the corresponding fragments from cDNA templates from Chinese kale. BP Clonase (Thermo Fisher Scientific, USA) was used to clone the resulting PCR products into the vector pDONR221. LR recombination reactions were subsequently performed between the resulting pENTR plasmids and the destination vector pGWB6 (35S promote, N-sGFP) [50].

In the VIGS experiment, 40 bp target oligonucleotides were selected in the highly conserved region of four *BoESP*s coding sequences. Two sets (pTY-S/BoESP-1 and pTY-S/BoESP-2) were synthesized to increase the efficiency of VIGS. We used the vector reported in a previous study [51]. The oligonucleotides were annealed and mixed with SnaBI-digested pTY-S plasmids to produce the working plasmids, namely pTY-BoESP-1 and pTY-BoESP-2.

### Transient expression in *Nicotiana benthamiana* and transformation in *Arabidopsis*

*N. benthamiana* plants were grown in the same growth chamber as Chinese kale sprouts. The *Agrobacterium* strain GV3101 was used as the carrier for transient expression. Bacterial suspensions carrying corresponding constructs were infiltrated into tobacco leaves according to the previous report [52]. After infiltration, *N. benthamiana* plants were placed in the growth chamber for 48 hours before observation under a confocal microscope.

*Arabidopsis* plants (Col-0) were grown at 21°C under a 16 h/8 h light/dark cycle with 70% relative humidity. The *Agrobacterium tumefaciens* strain GV3101 was employed for the transformation experiment. Selection of transformants was carried out according to the antibiotic markers associated with the corresponding vectors used. The root of transformants that heterologously overexpressing *BoESP*s was used for the observation under a confocal microscope.

### Virus-induced gene silencing of *BoESPs* in Chinese kale sprouts

The inoculum was prepared by adding pTY-BoESP-1 and pTY-BoESP-2 or empty plasmid pTY-S into infection media containing 10 mM MgCl_2_ and MES with a final concentration of 14 ng/μL. Germinated Chinese kale seeds with radicle protrusion through the seed coat were immersed in the inoculum and infiltrated via vacuuming for 30 seconds twice. The infiltrated seeds were then planted and grown under the same condition as samples for GSL detection. After 9 days, 10 sprouts were collected as one replicate to determine the expression level of *BoESP*s and the content of GBPs.

### RNA extraction and expression analysis

Total RNA was isolated by Trizol reagent and mRNA was reverse transcribed into cDNA according to the manufacturer’s instruction (Takara, Japan). The qPCR was conducted with CFX96 Real-Time PCR Detection System (Bio-Rad, USA). *β-ACTIN* was used as a housekeeping gene. The relative expression level of target genes was computed with the 2^−ΔΔCT^ method [53]. Gene-specific primer sequences for qPCR are listed in Table S6 (see online supplementary material).

### RNA-seq assay

Total RNA was extracted from seeds and 3 days old sprouts. NovaSeq 6000 platform (Illumina, USA) was used to sequence the sequencing library (Shanghai Personal Biotechnology Cp. Ltd, China). The filtered reads were mapped to the reference genome (Brassica_oleracea.BOL.dna.toplevel.fa, http://plants.ensembl.org/Brassica_oleracea/Info/Index) using HISAT2 v2.0.5.

### Determination of glucosinolates and glucosinolate breakdown products

Glucosinolates and glucosinolate breakdown products were extracted and determined as previously described with some modifications [4]. Glucosinolates were extracted from 20 mg samples by 2 mL of 90% methanol. Data were given as μmol/g or μmol/plant (the value of μmol/g times the weight of a single plant). For the detection of glucosinolate breakdown products, frozen sprouts (300 mg) were homogenized in liquid nitrogen. Data were given as μmol/g or μmol/plant (the value of μmol/g times the weight of a single plant).

### Determination of thiols

The detection of cysteine and glutathione was carried out as previously described [54]. In this study, about 300 mg of frozen sprouts were homogenized in liquid nitrogen.

### Statistical analysis

At least three biological replicates were taken for each experiment, and values were represented as the mean ± SD. The SPSS package program version 16.0 was used to do statistical analysis. Multiple comparisons were subjected to one-way ANOVA test and the least significant difference (LSD) test with a significant level of 5% (*P* < 0.05), while the values of *BoESP* expression in response to plant hormones were analysed by two-way ANOVA and Tukey’s test (*P* < 0.05). Pairwise comparisons were subjected to Student’s *t*-test (*P* < 0.05). Significance was indicated by asterisks * or different letters.

## Acknowledgements

We are grateful to Prof. Mingfang Zhang (Zhengjiang University, China) for kindly providing the plasmid pTY-S. This work was supported by National Natural Science Foundation of China (32172593), Natural Science Foundation of Zhejiang Province (LY21C020002), Science and Technology Plan Project of Ningbo City (2021Z132), and Zhejiang Province Commonweal Projects (NOLGN20C150009).

## Author contributions

Q.W. and H.M. conceived and designed the research. H.M., C.X., S.Y., J.W. and Y.Z. performed the experiments and analysed the data. H.M., C.X., and Q.W. wrote the manuscript. All authors read and approved the manuscript.

## Data availability

The datasets that support the findings of this study are available from the corresponding author upon reasonable request.

## Conflict of interest

The authors declare that they have no conflict of interest.

## Supplementary data

Supplementary data is available at *Horticulture Research* online.

## Supplementary Material

Web_Material_uhad029

## References

[1] Rajjou L , Duval M, Gallardo K et al. Seed germination and vigor. Annu Rev Plant Biol. 2012;63:507–33. 22136565 10.1146/annurev-arplant-042811-105550

[2] Gan RY , Wang MF, Lui WY et al. Dynamic changes in phytochemical composition and antioxidant capacity in green and black mung bean (*Vigna radiata*) sprouts. Int J Food Sci Technol. 2016;51:2090–8.

[3] Sun B , Liu N, Zhao Y et al. Variation of glucosinolates in three edible parts of Chinese kale (*Brassica alboglabra* Bailey) varieties. Food Chem. 2011;124:941–7.

[4] Zeng W , Tao H, Li Y et al. The flavor of Chinese kale sprouts is affected by genotypic variation of glucosinolates and their breakdown products. Food Chem. 2021;359:129824. 33965761 10.1016/j.foodchem.2021.129824

[5] Cole RA . Volatile components produced during ontogeny of some cultivated crucifers. J Sci Food Agr. 1980;31:549–57.

[6] Bellostas N , Kachlicki P, Sørensen JC et al. Glucosinolate profiling of seeds and sprouts of *B. oleracea* varieties used for food. Sci Hortic. 2007;114:234–42.

[7] Bhardwaj HL , Hamama AA. Accumulation of glucosinolate, oil, and erucic acid in developing brassica seeds. Ind Crop Prod. 2003;17:47–51.

[8] Malik MS , Riley MB, Norsworthy JK et al. Glucosinolate profile variation of growth stages of wild radish (*Raphanus raphanistrum*). J Agr Food Chem. 2010;58:3309–15. 20163113 10.1021/jf100258c

[9] Zhang T , Liu R, Zheng J et al. Insights into glucosinolate accumulation and metabolic pathways in *Isatis indigotica* Fort. BMC Plant Biol. 2022;22:78. 35193497 10.1186/s12870-022-03455-6 PMC8862337

[10] Palaniswamy UR , McAvoy RJ, Bible BB et al. Ontogenic variations of ascorbic acid and phenethyl isothiocyanate concentrations in watercress (*Nasturtium officinale* r.Br.) leaves. J Agr Food Chem. 2003;51:5504–9. 12926905 10.1021/jf034268w

[11] Brown PD , Tokuhisa JG, Reichelt M et al. Variation of glucosinolate accumulation among different organs and developmental stages of *Arabidopsis thaliana*. Phytochemistry. 2003;62:471–81. 12620360 10.1016/s0031-9422(02)00549-6

[12] McGregor DI . Glucosinolate content of developing rapeseed (*Brassica napus* L.'Midas') seedlings. Can J Plant Sci. 1988;68:367–80.

[13] Petersen B , Chen S, Hansen C et al. Composition and content of glucosinolates in developing Arabidopsis thaliana. Planta. 2002;214:562–71. 11925040 10.1007/s004250100659

[14] Wittstock U , Meier K, Dörr F et al. NSP-dependent simple nitrile formation dominates upon breakdown of major aliphatic Glucosinolates in roots, seeds, and seedlings of *Arabidopsis thaliana* Columbia-0. Front Plant Sci. 2016;7:1821. 27990154 10.3389/fpls.2016.01821 PMC5131009

[15] Burow M , Markert J, Gershenzon J et al. Comparative biochemical characterization of nitrile-forming proteins from plants and insects that alter myrosinase-catalysed hydrolysis of glucosinolates. FEBS J. 2006;273:2432–46. 16704417 10.1111/j.1742-4658.2006.05252.x

[16] Kissen R , Bones AM. Nitrile-specifier proteins involved in glucosinolate hydrolysis in Arabidopsis thaliana. J Biol Chem. 2009;284:12057–70. 19224919 10.1074/jbc.M807500200 PMC2673275

[17] Sikorska-Zimny K , Beneduce L. The glucosinolates and their bioactive derivatives in *Brassica*: a review on classification, biosynthesis and content in plant tissues, fate during and after processing, effect on the human organism and interaction with the gut microbiota. Crit Rev Food Sci. 2021;61:2544–71. 10.1080/10408398.2020.1780193 32584172

[18] Matusheski NV , Swarup R, Juvik JA et al. Epithiospecifier protein from broccoli (*Brassica oleracea* L. ssp. italica) inhibits formation of the anticancer aagent sulforaphane. J Agr Food Chem. 2006;54:2069–76. 16536577 10.1021/jf0525277

[19] Nastruzzi C , Cortesi R, Esposito E et al. In vitro antiproliferative activity of isothiocyanates and nitriles generated by myrosinase-mediated hydrolysis of glucosinolates from seeds of cruciferous vegetables. J. Agr. Food Chem. 2000;48:3572–5. 10956152 10.1021/jf000191p

[20] Matusheski NV , Jeffery EH. Comparison of the bioactivity of two glucoraphanin hydrolysis products found in broccoli, sulforaphane and sulforaphane nitrile. J Agr Food Chem. 2001;49:5743–9. 11743757 10.1021/jf010809a

[21] Wittstock U , Kliebenstein DJ, Lambrix V et al. Glucosinolate hydrolysis and its impact on generalist and specialist insect herbivores. In Romeo JT (Ed.), Integrative Phytochemistry: from Ethnobotany to Molecular Ecology. Amsterdam: Elsevier, 2003, 101–26.

[22] Shofran BG , Purrington ST, Breidt F et al. Antimicrobial properties of sinigrin and its hydrolysis products. J Food Sci. 1998;63:621–4.

[23] Harun S , Abdullah-Zawawi M, Goh H et al. A comprehensive gene inventory for glucosinolate biosynthetic pathway in Arabidopsis thaliana. J. Agr. Food Chem. 2020;68:7281–97. 32551569 10.1021/acs.jafc.0c01916

[24] Burow M , Rice M, Hause B et al. Cell- and tissue-specific localization and regulation of the epithiospecifier protein in Arabidopsis thaliana. Plant Mol Biol. 2007;64:173–85. 17390109 10.1007/s11103-007-9143-1

[25] Lambrix V , Reichelt M, Mitchell-Olds T et al. The Arabidopsis epithiospecifier protein promotes the hydrolysis of glucosinolates to nitriles and influences Trichoplusia ni herbivory. Plant Cell. 2001;13:2793–807. 11752388 10.1105/tpc.010261 PMC139489

[26] Zabala MT , Grant M, Bones AM et al. Characterisation of recombinant epithiospecifier protein and its over-expression in Arabidopsis thaliana. Phytochemistry. 2005;66:859–67. 15845404 10.1016/j.phytochem.2005.02.026

[27] Tookey HL . Crambe thioglucoside glucohydrolase (EC 3.2.3.1): separation of a protein required for epithiobutane formation. Can J Chem. 1973;51:1654–60. 10.1139/o73-222 4130022

[28] Zhang L , Cai X, Wu J et al. Improved *Brassica rapa* reference genome by single-molecule sequencing and chromosome conformation capture technologies. Hortic Res. 2018;5:50. 30131865 10.1038/s41438-018-0071-9 PMC6092429

[29] Bernardi R , Negri A, Ronchi S et al. Isolation of the epithiospecifier protein from oil-rape (*Brassica napus* ssp. oleifera) seed and its characterization. FEBS Lett. 2000;467:296–8. 10675557 10.1016/s0014-5793(00)01179-0

[30] Foo HL , Grønning LM, Goodenough L et al. Purification and characterisation of epithiospecifier protein from *Brassica napus*: enzymic intramolecular sulphur addition within alkenyl thiohydroximates derived from alkenyl glucosinolate hydrolysis. FEBS Lett. 2000;468:243–6. 10692595 10.1016/s0014-5793(00)01176-5

[31] Witzel K , Abu Risha M, Albers P et al. Identification and characterization of three epithiospecifier protein isoforms in *Brassica oleracea*. Front Plant Sci. 2019;10. 10.3389/fpls.2019.01552 PMC6930892 31921230

[32] Frerigmann H , Gigolashvili T. Update on the role of R2R3-MYBs in the regulation of glucosinolates upon sulfur deficiency. Front Plant Sci. 2014;5:626. 25426131 10.3389/fpls.2014.00626 PMC4224069

[33] Sugiyama R , Li R, Kuwahara A et al. Retrograde sulfur flow from glucosinolates to cysteine in *Arabidopsis thaliana*. P Natl Acad Sci USA. 2021;118:e2017890118. 10.1073/pnas.2017890118 PMC8179156 34035165

[34] Aarabi F , Naake T, Fernie AR et al. Coordinating sulfur pools under sulfate deprivation. Trends Plant Sci. 2020;25:1227–39. 32800669 10.1016/j.tplants.2020.07.007

[35] Sikorska-Zimny K , Beneduce L. The metabolism of glucosinolates by gut microbiota. Nutrients. 2021;13:2750. 34444909 10.3390/nu13082750 PMC8401010

[36] Gu Y , Guo Q, Zhang L et al. Physiological and biochemical metabolism of germinating broccoli seeds and sprouts. J. Agr. Food Chem. 2012;60:209–13. 22142148 10.1021/jf203599v

[37] Williams DJ , Critchley C, Pun S et al. Epithiospecifier protein activity in broccoli: the link between terminal alkenyl glucosinolates and sulphoraphane nitrile. Phytochemistry. 2008;69:2765–73. 18977005 10.1016/j.phytochem.2008.09.018

[38] Romero LC , Aroca MÁ, Laureano-Marín AM et al. Cysteine and cysteine-related signaling pathways in Arabidopsis thaliana. Mol Plant. 2014;7:264–76. 24285094 10.1093/mp/sst168

[39] Crawford N , Kahn M, Leustek T. Nitrogen and sulfur. In: Buchanan B, Cruissem W, Jones R, eds. 826 Biochemistry and Molecular Biology of Plants. Society of Plant Physiologists, Maryland, 2000.

[40] Andernach L , Witzel K, Hanschen FS. Glucosinolate-derived amine formation in Brassica oleracea vegetables. Food Chem. 2023;405:134907. 36417803 10.1016/j.foodchem.2022.134907

[41] Hanschen FS , Schreiner M. Isothiocyanates, nitriles, and epithionitriles from glucosinolates are affected by genotype and developmental stage in Brassica oleracea varieties. Front Plant Sci. 2017;8:1095. 28690627 10.3389/fpls.2017.01095 PMC5479884

[42] Wiesner M , Zrenner R, Krumbein A et al. Genotypic variation of the Glucosinolate profile in Pak Choi (*Brassica rapa* ssp.chinensis). J. Agr. Food Chem. 2013;61:1943–53. 23350944 10.1021/jf303970k

[43] Miao Y , Zentgraf U. The antagonist function of Arabidopsis WRKY53 and ESR/ESP in leaf senescence is modulated by the jasmonic and salicylic acid equilibrium. Plant Cell. 2007;19:819–30. 17369373 10.1105/tpc.106.042705 PMC1867371

[44] Augustine R , Bisht NC. Biotic elicitors and mechanical damage modulate glucosinolate accumulation by co-ordinated interplay of glucosinolate biosynthesis regulators in polyploid *Brassica juncea*. Phytochemistry. 2015;117:43–50. 26057228 10.1016/j.phytochem.2015.05.015

[45] Barco B , Clay NK. Evolution of glucosinolate diversity via whole-genome duplications, gene rearrangements, and substrate promiscuity. Annu Rev Plant Biol. 2019;70:585–604. 31035830 10.1146/annurev-arplant-050718-100152

[46] Kuchernig JC , Burow M, Wittstock U. Evolution of specifier proteins in glucosinolate-containing plants. BMC Evol Biol. 2012;12:127–7. 22839361 10.1186/1471-2148-12-127 PMC3482593

[47] Wang Z , Yang R, Guo L et al. Effects of abscisic acid on glucosinolate content, isothiocyanate formation and myrosinase activity in cabbage sprouts. Int J Food Sci Tech. 2015;50:1839–46.

[48] Miao HY , Wang MY, Chang JQ et al. Effects of glucose and gibberellic acid on glucosinolate content and antioxidant properties of Chinese kale sprouts. J Zhejiang Univ Sci B. 2017;18:1093–100. 29204989 10.1631/jzus.B1700308 PMC5742292

[49] Chen C , Chen H, Zhang Y et al. TBtools: An Integrative Toolkit Developed for Interactive Analyses of Big Biological Data. Mol Plant. 2020;13:1194–202. 32585190 10.1016/j.molp.2020.06.009

[50] Nakagawa TSUM et al. Improved gateway binary vectors: high-performance vectors for creation of fusion constructs in transgenic analysis of plants. Biosci Biotechnol Biochem. 2007;71:2095–100. 17690442 10.1271/bbb.70216

[51] Jupin I . A protocol for VIGS in Arabidopsis thaliana using a one-step TYMV-derived vector. Methods Mol Biol. 2013;975:197–210. 23386305 10.1007/978-1-62703-278-0_15

[52] Ho CM , Lee YR, Kiyama LD et al. Arabidopsis microtubule-associated protein MAP65-3 cross-links antiparallel microtubules toward their plus ends in the phragmoplast via its distinct C-terminal microtubule binding domain. Plant Cell. 2012;24:2071–85. 22570443 10.1105/tpc.111.092569 PMC3442588

[53] Livak KJ , Schmittgen TD. Analysis of relative gene expression data using real-time quantitative pcr and the 2−ΔΔCT method. Methods. 2001;25:402–8. 11846609 10.1006/meth.2001.1262

[54] Miao H , Cai C, Wei J et al. Glucose enhances indolic glucosinolate biosynthesis without reducing primary sulfur assimilation. Sci Rep. 2016;6:31854–4. 27549907 10.1038/srep31854 PMC4994012

