# Supplementary material for: Enhancing health-promoting isothiocyanates in Chinese kale sprouts via manipulating BoESP
Source: Hortic Res. 2023 Feb 21;10(4):uhad029. doi: 10.1093/hr/uhad029 (PMC10117428; doi:10.1093/hr/uhad029)
Supplement: Web_Material_uhad029 [file web_material_uhad029.zip › Miao_et_al_Supplementary data-revised-round3.docx]

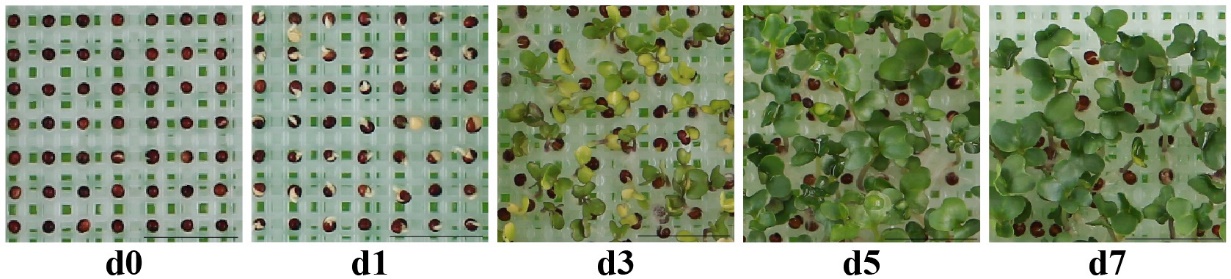


Supplementary Figure 1 Images of 0-d-, 1-d-, 3-d-, 5-d-, and 7-d-old germinated Chinese kale sprouts. Bar=2 cm.


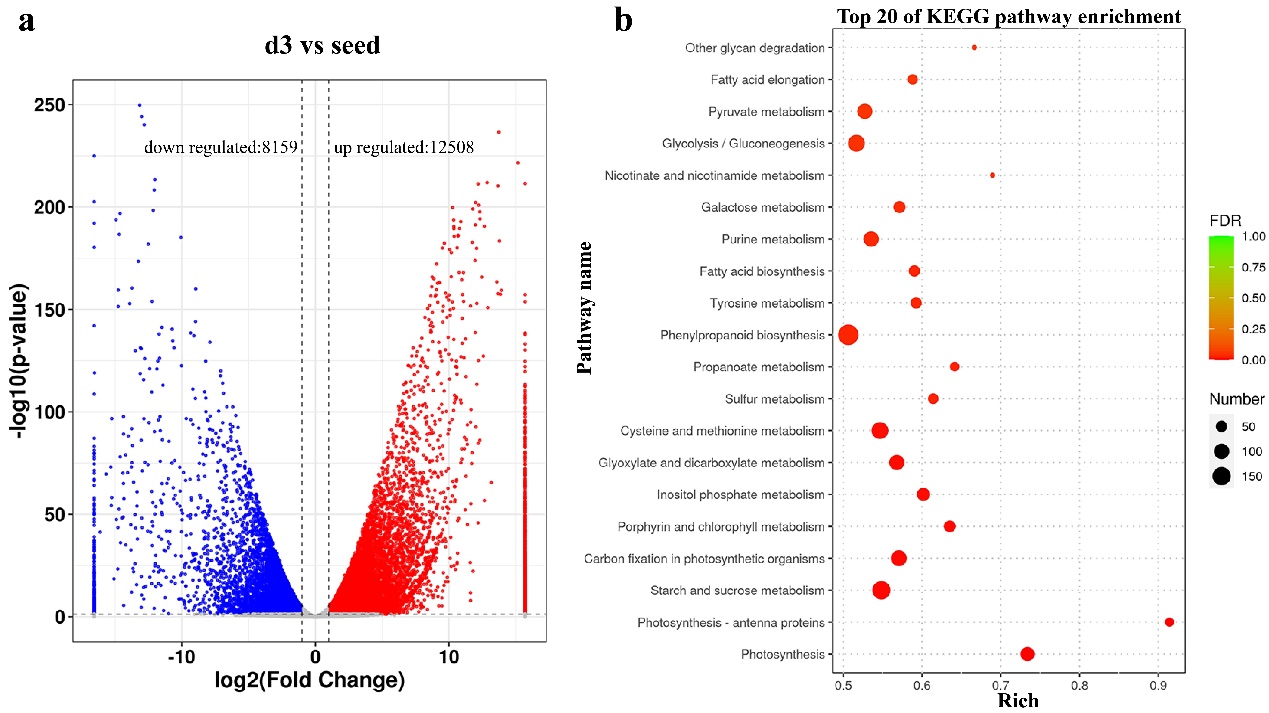


Supplementary Figure 2 Volcano plot of differentially expressed genes in seed vs d3 (A) and KEGG enrichment analysis (B).


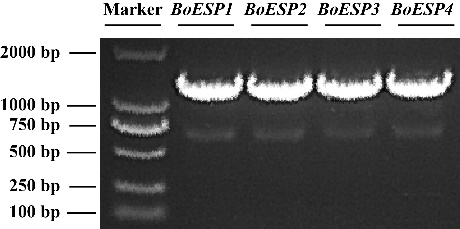


Supplementary Figure 3 **Gel electrophoresis image of** *BoESP* coding sequence.


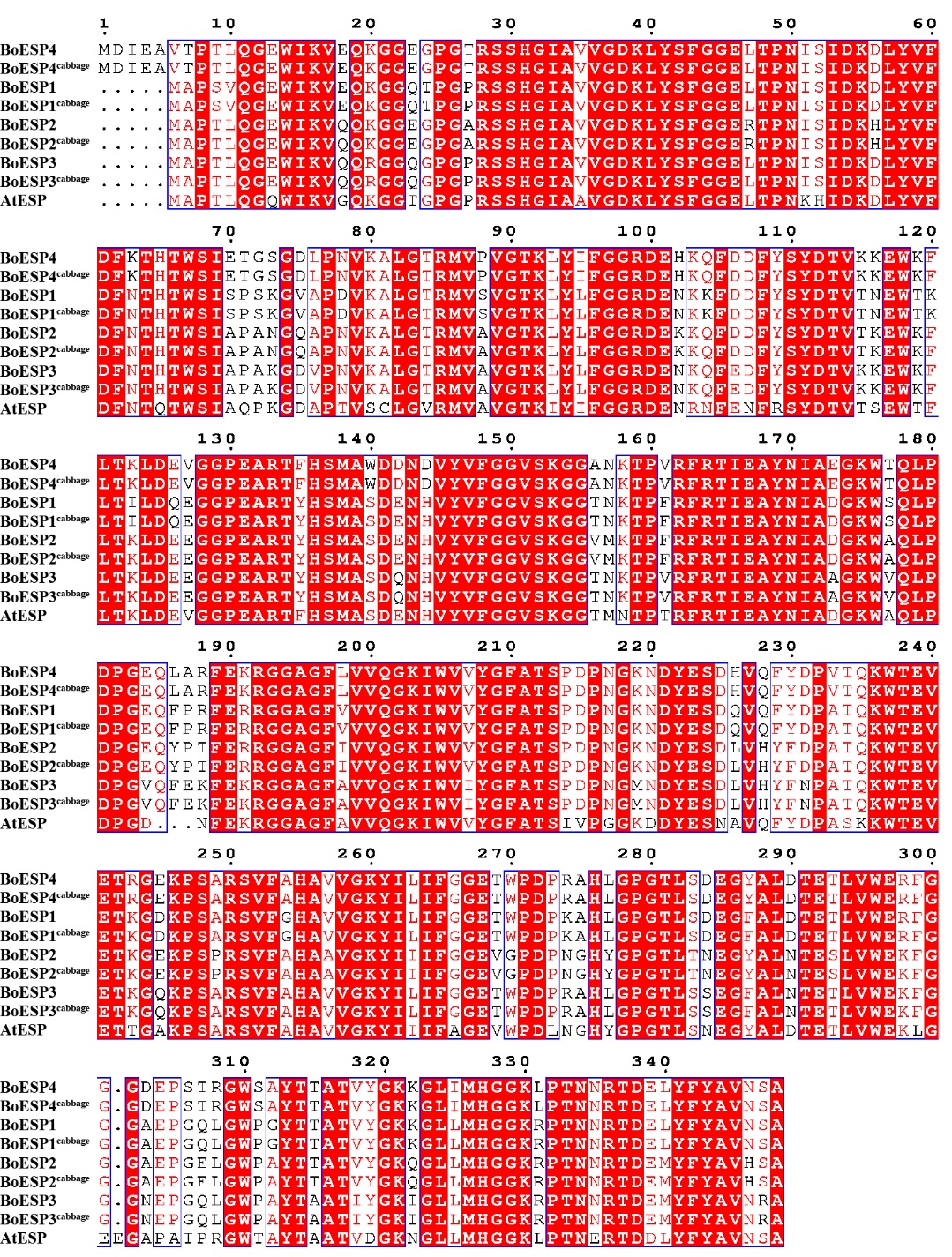


Supplementary Figure 4 Amino acid sequence alignment of AtESP, BoESP and BoESP^cabbage^.


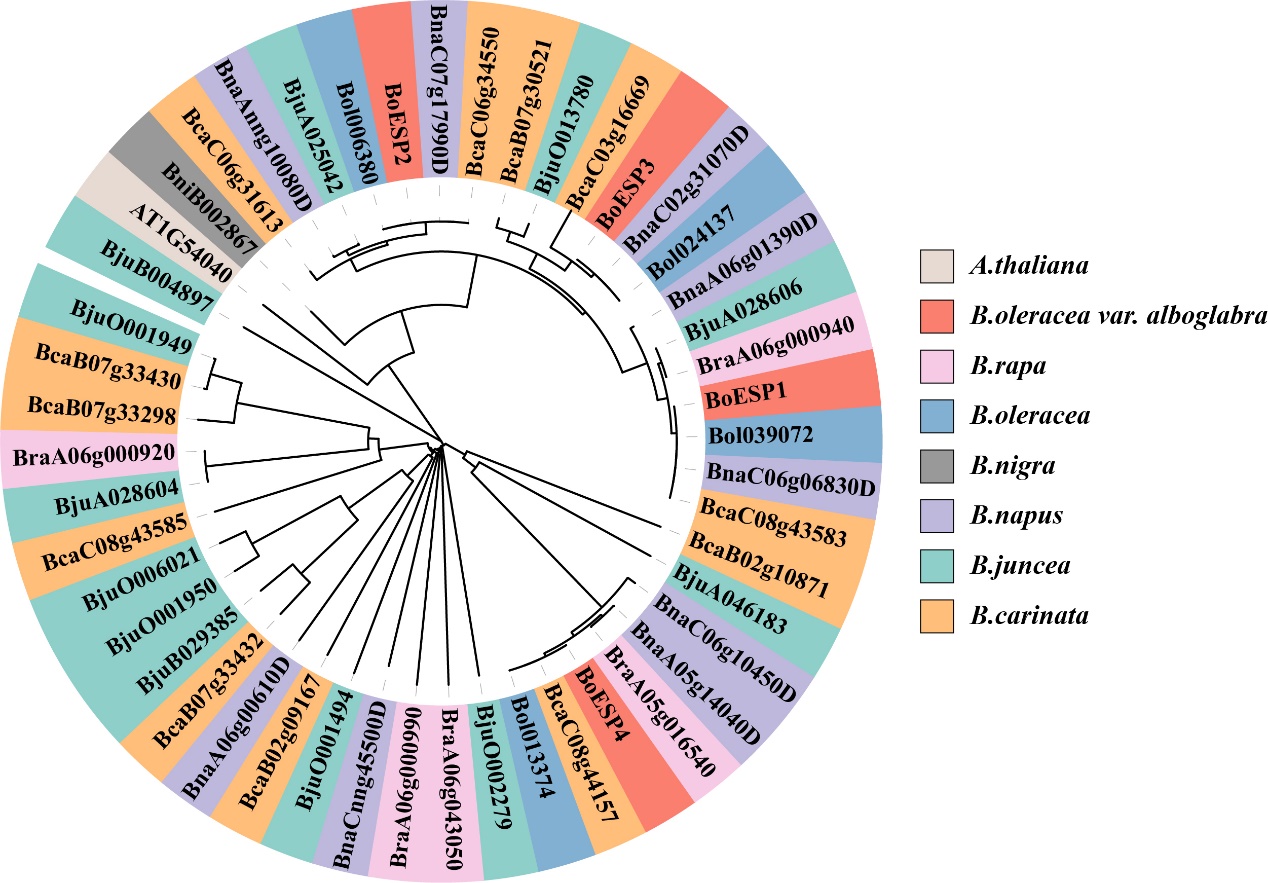


Supplementary Figure 5 Evolution analysis of epithiospecifier protein in *A.thaliana*, *B.oleracea var. alboglabra* and six species in U’s triangle.


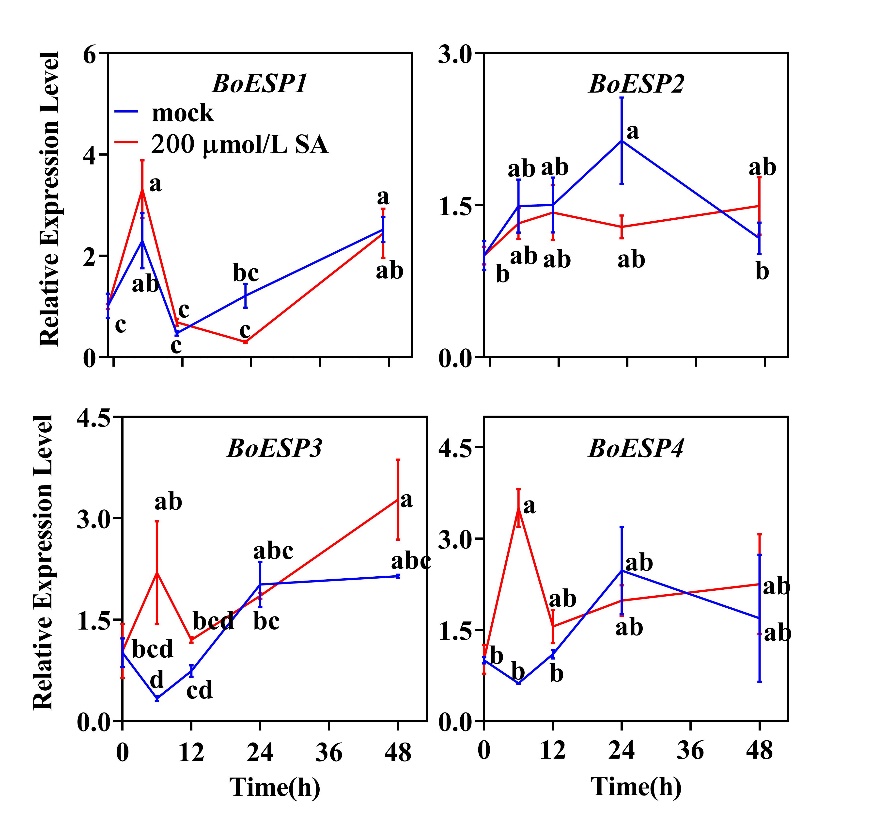


Supplementary Figure 6 Effect of SA on the expression of *BoESP*s in Chinese kale sprouts.

Supplementary Table 1 Nomenclature of glucosinolates (GLSs) and their corresponding GLS breakdown products detected in Chinese kale sprouts

|  |  | Glucosinolates (GLS) | |  | Corresponding breakdown product (GBP) | | | | | | | | | | |  |
| --- | --- | --- | --- | --- | --- | --- | --- | --- | --- | --- | --- | --- | --- | --- | --- | --- |
| Groups | | Abbreviation | Chemical name of side chain (trivial name) |  | Isothiocyanate (ITC) | |  | Nitrile (NIT) | |  | | Epithionitrile (EPT) | | |  | |
|  |  |  |  | | Abbreviation | Name |  | Abbreviation | Name | |  | Abbreviation | Name | |  | |
| Aliphatic  (AGS) | Alkenyl | SIN | 2-Propenyl (sinigrin) | | 2Prop-ITC | 2-Propenyl ITC | | 2Prop-CN | 3-Butenylnitrile | | | CETP | | 1-Cyano-2,3-epithiopropane | |  |
|  |  | PRO | 2-(R)-2-hydroxy-3-butenyl (progoitrin) | | OZT | 5-Vinyl-1,3-oxazolidine-2-thione | | n.d. | n.d. | | | CHETB | | 3-Hydroxy-4,5-epithiopentane | |  |
|  |  | GNL | 2-Hydroxy-4-pentenyl (gluconapoleiferin) | | n.d. | n.d. | | n.d. | n.d. | | | n.d. | | n.d. | |  |
|  |  | GNA | 3-Butenyl (gluconapin) | | 3But-ITC | 3-Butenyl ITC | | 3But-CN | 4-Pentenenitrile | | | CETB | | 1-Cyano-3,4-epithiobutane | |  |
|  | Alkyl | GIB | 3-(Methylsulphinyl)propyl (glucoiberin) | | 3MSOP-ITC | 3-(Methylsulphinyl)propyl ITC | | n.d. | n.d. | | |  | |  | |  |
|  |  | GIV | 3-(Methylthio)propyl (glucoiberverin) | | 3MTP-ITC | 3-(Methylthio)propyl ITC | | 3MTP-CN | 4-(Methylthio)butanenitrile | | |  | |  | |  |
|  |  | GRA | 4-(Methylsulfinyl)butyl (glucoraphanin) | | 4MSOB-ITC | 4-(Methylsulfinyl)butyl ITC | | 4MSOB-CN | 5-(Methylsulfinyl)pentanenitrile | | |  | |  | |  |
|  |  | GER | 4-(Methylthio)butyl (glucoerucin) | | 4MTB-ITC | 4-(Methylthio)butyl ITC | | 4MTB-CN | 5-(Methylthio)pentanenitrile | | |  | |  | |  |
| Indole  (IGS) |  | NGBS | 1-Methoxy-3-indolylmethyl (neoglucobrassicin) | | n.d. | n.d. | | n.d. | n.d. | | |  | |  | |  |
|  |  | 4-OMGBS | 4-Methoxy-3-indolylmethyl (4-methoxyglucobrassicin) | | n.d. | n.d. | | n.d. | n.d. | | |  | |  | |  |
|  |  | 4-OHGBS | 4-Hydroxy-3-indolylmethyl (4-hydroxyglucobrassicin) | | n.d. | n.d. | | n.d. | n.d. | | |  | |  | |  |
|  |  | GBS | 3-Indolylmethyl (glucobrassicin) | | n.d. | n.d. | | n.d. | n.d. | | |  | |  | |  |

n.d., not detected

Supplementary Table 2 The content of GSL breakdown products in Chinese kale at different developmental stages (μmol/g FW).

| Name | Data type | seed | d0 | d1 | d3 | d5 | d7 |
| --- | --- | --- | --- | --- | --- | --- | --- |
| 2Prop-CN | mean | 0.3365 | 0.9579 | 0.6416 | 0.3690 | 0.2272 | 0.2005 |
|  | SD | 0.0275 | 0.0417 | 0.0717 | 0.0289 | 0.0126 | 0.0044 |
| 3But-CN | mean | 1.6955 | 5.0839 | 3.4781 | 1.0127 | 0.5361 | 0.4052 |
|  | SD | 0.1324 | 0.1819 | 0.3281 | 0.0572 | 0.0123 | 0.0043 |
| 2Prop-ITC | mean | 2.5121 | 2.0458 | 2.1422 | 1.5793 | 0.0765 | 0.0788 |
|  | SD | 0.1519 | 0.0971 | 0.2191 | 0.0732 | 0.0062 | 0.0022 |
| 3But-ITC | mean | 8.7100 | 7.5498 | 7.0661 | 5.0692 | 0.5390 | 0.4090 |
|  | SD | 0.4462 | 0.3600 | 0.8039 | 0.2273 | 0.0150 | 0.0206 |
| CETP | mean | 0.1835 | 3.0481 | 3.1326 | 2.3806 | 2.4579 | 1.7353 |
|  | SD | 0.0044 | 0.0286 | 0.3418 | 0.1595 | 0.1102 | 0.0407 |
| 3MTP-CN | mean | 0.0729 | 0.4408 | 0.4087 | 0.1571 | 0.1624 | 0.0950 |
|  | SD | 0.0031 | 0.0048 | 0.0355 | 0.0107 | 0.0082 | 0.0018 |
| CETB | mean | 0.6076 | 15.5848 | 14.5990 | 6.7853 | 6.4752 | 4.2575 |
|  | SD | 0.0384 | 0.0976 | 1.5551 | 0.4170 | 0.3426 | 0.0773 |
| 4MTB-CN | mean | 0.2521 | 1.8345 | 1.3080 | 0.3989 | 0.5522 | 0.3357 |
|  | SD | 0.0133 | 0.0421 | 0.1372 | 0.0179 | 0.0348 | 0.0059 |
| 3MTP-ITC | mean | 0.1615 | 0.2244 | 0.3199 | 0.1052 | 0.0898 | 0.0899 |
|  | SD | 0.0150 | 0.0118 | 0.0344 | 0.0105 | 0.0066 | 0.0012 |
| 4MTB-ITC | mean | 0.1274 | 0.3748 | 0.3465 | 0.1782 | 0.1764 | 0.1281 |
|  | SD | 0.0031 | 0.0149 | 0.0352 | 0.0089 | 0.0058 | 0.0065 |
| OZT | mean | 0.0936 | 0.2233 | 0.2270 | 0.1713 | 0.1802 | 0.1735 |
|  | SD | 0.0056 | 0.0202 | 0.0272 | 0.0062 | 0.0190 | 0.0148 |
| CHETB | mean | 0.4331 | 0.5780 | 0.5519 | 0.4013 | 0.4663 | 0.4889 |
|  | SD | 0.0233 | 0.0621 | 0.0468 | 0.0398 | 0.0668 | 0.0406 |
| 4MSOB-CN | mean | 0.1090 | 0.1283 | 0.1200 | 0.0964 | 0.1339 | 0.1456 |
|  | SD | 0.0081 | 0.0176 | 0.0146 | 0.0075 | 0.0051 | 0.0052 |
| 3MSOP-ITC | mean | 0.4269 | 1.5068 | 1.4410 | 0.5240 | 0.6597 | 0.5081 |
|  | SD | 0.0143 | 0.0491 | 0.1375 | 0.0354 | 0.0227 | 0.0160 |
| 4MSOB-ITC | mean | 0.5813 | 1.2364 | 1.2598 | 0.5776 | 0.1028 | 0.0679 |
|  | SD | 0.0212 | 0.1386 | 0.1314 | 0.0376 | 0.0226 | 0.0025 |

2Prop-CN: 3-butenyl nitrile; 3But-CN: 4-pentenenitrile; 2Prop-ITC: 2-propenyl ITC; 3But-ITC: 3-butenyl ITC; CETP: 1-cyano-2,3-epithiopropane; 3MTP-CN: 4-(methylthio)butanenitrile; CETB: 1-cyano-3,4-epithiobutane; 4MTB-CN: 5-(methylthio)pentanenitrile; 3MTP-ITC: 3-(methylthio)propyl ITC; 4MTB-ITC: 4-(methylthio)butyl ITC; OZT: 5-vinyl-1,3-oxazolidine-2-thione; CHETB: 3-hydroxy-4,5-epithiopentane; 4MSOB-CN: 5-(methylsulfinyl)pentanenitrile; 3MSOP-ITC: 3-(methylsulphinyl)propyl ITC; 4MSOB-ITC: 4-(methylsulfinyl)butyl ITC.

Supplementary Table 3 The content of GSLs in Chinese kale at different developmental stages (μmol/g DW).

| Name | Data type | seed | d0 | d1 | d3 | d5 | d7 |
| --- | --- | --- | --- | --- | --- | --- | --- |
| GIB | mean | 4.1086 | 3.8960 | 6.8424 | 4.7922 | 4.9122 | 5.1018 |
|  | SD | 1.6918 | 1.1224 | 1.5106 | 1.0870 | 0.5726 | 0.9742 |
| PRO | mean | 2.7016 | 1.0490 | 1.5622 | 7.8578 | 9.5572 | 10.2444 |
|  | SD | 3.2286 | 0.2496 | 0.4988 | 0.3978 | 0.4390 | 0.5782 |
| SIN | mean | 12.4658 | 10.4134 | 13.5558 | 17.3102 | 17.9676 | 20.6694 |
|  | SD | 4.1548 | 0.8236 | 0.4162 | 0.9228 | 0.8640 | 1.2012 |
| GRA | mean | 8.4968 | 7.9810 | 11.7988 | 8.3790 | 8.6854 | 8.7218 |
|  | SD | 3.1150 | 1.9254 | 2.4614 | 1.9464 | 1.0382 | 1.7494 |
| GNA | mean | 42.3002 | 34.3212 | 37.4924 | 42.8910 | 39.9218 | 44.7588 |
|  | SD | 7.3532 | 4.9538 | 1.6772 | 1.7054 | 2.4294 | 2.3656 |
| GIV | mean | 1.8746 | 1.5396 | 2.8424 | 2.1714 | 2.8670 | 3.1012 |
|  | SD | 0.6120 | 0.1472 | 0.8332 | 0.8474 | 0.5792 | 0.1508 |
| 4-OHGBS | mean | 0.7856 | 0.6442 | 0.6790 | 2.3476 | 1.8806 | 1.5634 |
|  | SD | 0.1762 | 0.0512 | 0.0920 | 0.4208 | 0.0638 | 0.2458 |
| GER | mean | 9.7990 | 7.0450 | 12.3364 | 12.2306 | 11.7324 | 10.7426 |
|  | SD | 2.9364 | 0.9024 | 2.2814 | 0.4458 | 0.5470 | 0.6046 |
| GBS | mean | 0.0510 | 0.0542 | 0.0832 | 0.5000 | 0.7888 | 0.9084 |
|  | SD | 0.0242 | 0.0100 | 0.0196 | 0.0334 | 0.0382 | 0.0776 |
| 4-OMGBS | mean | 0.0790 | 0.0886 | 0.0952 | 1.1368 | 2.2078 | 3.6136 |
|  | SD | 0.0196 | 0.0270 | 0.0144 | 0.1220 | 0.1020 | 0.3638 |
| GNL | mean | 0.3218 | 0.2014 | 0.2648 | 0.3262 | 0.3576 | 0.4060 |
|  | SD | 0.0552 | 0.0262 | 0.0314 | 0.0178 | 0.0248 | 0.0200 |
| NGBS | mean | 0.0472 | 0.0544 | 0.0970 | 0.7622 | 1.4776 | 2.7620 |
|  | SD | 0.0156 | 0.0104 | 0.0140 | 0.0282 | 0.0706 | 0.1626 |

GIB: glucoiberin; PRO: progoitrin; SIN: sinigrin; GRA: glucoraphanin; GNA: gluconapin; GIV: glucoiberverin; 4-OHGBS: 4-hydroxyglucobrassicin; GER: glucoerucin; GBS: glucobrassicin; 4-OMGBS: 4-methoxyglucobrassicin; GNL: gluconapoleiferin; NGBS: neoglucobrassicin.

Supplementary Table 4 Primers used in this study.

| Primer | Sequence (5’-3’) |
| --- | --- |
| β-BoACTIN-F | CCAGAGGTCTTGTTCCAGCCATC |
| β-BoACTIN-R | GTTCCACCACTGAGCACAATGTTAC |
| qPCR-BoESP1-F | CAAGGCGAGTGGATCAAGGT |
| qPCR-BoESP1-R | CTTGACGTCAGGGGCTACTC |
| qPCR-BoESP2-F | ATCCTGGTGAGCAGTACCCT |
| qPCR-BoESP2-R | AGTCTCCACTTCGGTCCACT |
| qPCR-BoESP3-F | GACGAACAAAACCCCTGTGC |
| qPCR-BoESP3-R | GATCAGGCGAAGTCGCAAAC |
| qPCR-BoESP4-F | AAGGCGAGTGGATCAAGGTG |
| qPCR-BoESP4-R | CCTGGTGCCTAAGGCTTTGA |
| BoESP1F | ATGGCTCCGAGTGTGCAAG |
| BoESP1R | CGCGGAATTAACTGCGTAGA |
| BoESP2F | ATGGCTCCCACATTGCAAGG |
| BoESP2R | GGCGGAATGGACCGC |
| BoESP3F | ATGGCTCCGACTTTGCAAGGC |
| BoESP3R | GGCGCGATTGACTGCGTAGAAG |
| BoESP4F | ATGGATATTGAAGCTGTGACTCC |
| BoESP4R | CGCGGAATTGACTGCGTAGA |
| attB1-BoESP1-F | GGGGACAAGTTTGTACAAAAAAGCAGGCTTCATGGCTCCGAGTGTGCAAG |
| attB2-BoESP1-R | GGGGACCACTTTGTACAAGAAAGGTGGGTCGCGGAATTAACTGCGTAGA |
| attB1-BoESP2-F | GGGGACAAGTTTGTACAAAAAAGCAGGCTTCATGGCTCCCACATTGCAAGG |
| attB2-BoESP2-R | GGGGACCACTTTGTACAAGAAAGGTGGGTGGCGGAATGGACCGC |
| attB1-BoESP3-F | GGGGACAAGTTTGTACAAAAAAGCAGGCTTCATGGCTCCGACTTTGCAAGGC |
| attB2-BoESP3-R | GGGGACCACTTTGTACAAGAAAGGTGGGTGGCGCGATTGACTGCGTAGAAG |
| attB1-BoESP4-F | GGGGACAAGTTTGTACAAAAAAGCAGGCTTCATGGATATTGAAGCTGTGACTCC |
| attB2-BoESP4-R | GGGGACCACTTTGTACAAGAAAGGTGGGTCGCGGAATTGACTGCGTAGA |
| pTY-S/BoESP-1 | TTGACTTCAACACTCACACTTGGTCAATCGCTCCAGCCAATTGGCTGGAGCGATTGACCAAGTGTGAGTGTTGAAGTCAA |
| pTY-S/BoESP-2 | TTGACTTCAAAACCCACACTTGGTCGATCGAAACGGGCAGCTGCCCGTTTCGATCGACCAAGTGTGGGTTTTGAAGTCAA |

Supplementary Table 5 Detailed information of cis-elements identified in the promoter region of all four *BoESP*s ^cabbage^.

| Target gene | Name | Motif_sequence | Location | Chain | Short_function |
| --- | --- | --- | --- | --- | --- |
| *BoESP1*^cabbage^ | AuxRR-core | GGTCCAT | 74 | - | cis-acting regulatory element involved in auxin responsiveness |
| *BoESP1*^cabbage^ | TCT-motif | TCTTAC | 86 | - | part of a light responsive element |
| *BoESP1*^cabbage^ | AuxRR-core | GGTCCAT | 237 | - | cis-acting regulatory element involved in auxin responsiveness |
| *BoESP1*^cabbage^ | TCT-motif | TCTTAC | 249 | - | part of a light responsive element |
| *BoESP1*^cabbage^ | AuxRR-core | GGTCCAT | 400 | - | cis-acting regulatory element involved in auxin responsiveness |
| *BoESP1*^cabbage^ | TCT-motif | TCTTAC | 412 | - | part of a light responsive element |
| *BoESP1*^cabbage^ | AuxRR-core | GGTCCAT | 563 | - | cis-acting regulatory element involved in auxin responsiveness |
| *BoESP1*^cabbage^ | TCT-motif | TCTTAC | 575 | - | part of a light responsive element |
| *BoESP1*^cabbage^ | AuxRR-core | GGTCCAT | 726 | - | cis-acting regulatory element involved in auxin responsiveness |
| *BoESP1*^cabbage^ | TCT-motif | TCTTAC | 738 | - | part of a light responsive element |
| *BoESP1*^cabbage^ | AuxRR-core | GGTCCAT | 889 | - | cis-acting regulatory element involved in auxin responsiveness |
| *BoESP1*^cabbage^ | TCT-motif | TCTTAC | 901 | - | part of a light responsive element |
| *BoESP1*^cabbage^ | TCT-motif | TCTTAC | 1064 | - | part of a light responsive element |
| *BoESP1*^cabbage^ | TATC-box | TATCCCA | 1090 | + | cis-acting element involved in gibberellin-responsiveness |
| *BoESP1*^cabbage^ | AuxRR-core | GGTCCAT | 1215 | - | cis-acting regulatory element involved in auxin responsiveness |
| *BoESP1*^cabbage^ | ACE | CTAACGTATT | 1233 | + | cis-acting element involved in light responsiveness |
| *BoESP1*^cabbage^ | ATCT-motif | AATCTAATCC | 1238 | - | part of a conserved DNA module involved in light responsiveness |
| *BoESP1*^cabbage^ | GA-motif | ATAGATAA | 1376 | + | part of a light responsive element |
| *BoESP1*^cabbage^ | TC-rich repeats | ATTCTCTAAC | 1387 | - | cis-acting element involved in defense and stress responsiveness |
| *BoESP1*^cabbage^ | Box 4 | ATTAAT | 1432 | - | part of a conserved DNA module involved in light responsiveness |
| *BoESP1*^cabbage^ | circadian | CAAAGATATC | 1556 | + | cis-acting regulatory element involved in circadian control |
| *BoESP1*^cabbage^ | AT-rich sequence | TAAAATACT | 1584 | - | element for maximal elicitor-mediated activation (2copies) |
| *BoESP1*^cabbage^ | TCT-motif | TCTTAC | 1912 | + | part of a light responsive element |
| *BoESP2*^cabbage^ | ARE | AAACCA | 25 | + | cis-acting regulatory element essential for the anaerobic induction |
| *BoESP2*^cabbage^ | ARE | AAACCA | 107 | - | cis-acting regulatory element essential for the anaerobic induction |
| *BoESP2*^cabbage^ | Sp1 | GGGCGG | 204 | - | light responsive element |
| *BoESP2*^cabbage^ | CGTCA-motif | CGTCA | 212 | + | cis-acting regulatory element involved in the MeJA-responsiveness |
| *BoESP2*^cabbage^ | TGACG-motif | TGACG | 212 | - | cis-acting regulatory element involved in the MeJA-responsiveness |
| *BoESP2*^cabbage^ | ABRE | ACGTG | 338 | - | cis-acting element involved in the abscisic acid responsiveness |
| *BoESP2*^cabbage^ | G-box | TACGTG | 338 | - | cis-acting regulatory element involved in light responsiveness |
| *BoESP2*^cabbage^ | ARE | AAACCA | 353 | - | cis-acting regulatory element essential for the anaerobic induction |
| *BoESP2*^cabbage^ | ABRE | CACGTG | 361 | + | cis-acting element involved in the abscisic acid responsiveness |
| *BoESP2*^cabbage^ | G-box | CACGTG | 361 | + | cis-acting regulatory element involved in light responsiveness |
| *BoESP2*^cabbage^ | G-Box | CACGTG | 361 | + | cis-acting regulatory element involved in light responsiveness |
| *BoESP2*^cabbage^ | ABRE | ACGTG | 362 | + | cis-acting element involved in the abscisic acid responsiveness |
| *BoESP2*^cabbage^ | GT1-motif | GGTTAA | 401 | + | light responsive element |
| *BoESP2*^cabbage^ | Box 4 | ATTAAT | 927 | + | part of a conserved DNA module involved in light responsiveness |
| *BoESP2*^cabbage^ | MBS | CAACTG | 1222 | + | MYB binding site involved in drought-inducibility |
| *BoESP2*^cabbage^ | GT1-motif | GGTTAA | 1589 | + | light responsive element |
| *BoESP2*^cabbage^ | ARE | AAACCA | 1602 | + | cis-acting regulatory element essential for the anaerobic induction |
| *BoESP2*^cabbage^ | GA-motif | ATAGATAA | 1610 | - | part of a light responsive element |
| *BoESP2*^cabbage^ | TC-rich repeats | ATTCTCTAAC | 1707 | + | cis-acting element involved in defense and stress responsiveness |
| *BoESP2*^cabbage^ | TGA-element | AACGAC | 1734 | - | auxin-responsive element |
| *BoESP3*^cabbage^ | TCA-element | CCATCTTTTT | 302 | + | cis-acting element involved in salicylic acid responsiveness |
| *BoESP3*^cabbage^ | ATCT-motif | AATCTAATCC | 553 | - | part of a conserved DNA module involved in light responsiveness |
| *BoESP3*^cabbage^ | AE-box | AGAAACTT | 597 | - | part of a module for light response |
| *BoESP3*^cabbage^ | MRE | AACCTAA | 819 | - | MYB binding site involved in light responsiveness |
| *BoESP3*^cabbage^ | Box 4 | ATTAAT | 847 | + | part of a conserved DNA module involved in light responsiveness |
| *BoESP3*^cabbage^ | GATA-motif | AAGGATAAGG | 913 | - | part of a light responsive element |
| *BoESP3*^cabbage^ | P-box | CCTTTTG | 986 | + | gibberellin-responsive element |
| *BoESP3*^cabbage^ | CGTCA-motif | CGTCA | 1292 | + | cis-acting regulatory element involved in the MeJA-responsiveness |
| *BoESP3*^cabbage^ | TGACG-motif | TGACG | 1292 | - | cis-acting regulatory element involved in the MeJA-responsiveness |
| *BoESP3*^cabbage^ | RY-element | CATGCATG | 1351 | - | cis-acting regulatory element involved in seed-specific regulation |
| *BoESP3*^cabbage^ | MSA-like | (T/C)C(T/C)AACGG(T/C)(T/C)A | 1564 | - | cis-acting element involved in cell cycle regulation |
| *BoESP3*^cabbage^ | MBS | CAACTG | 1589 | - | MYB binding site involved in drought-inducibility |
| *BoESP3*^cabbage^ | ABRE | AACCCGG | 1612 | + | cis-acting element involved in the abscisic acid responsiveness |
| *BoESP3*^cabbage^ | TCT-motif | TCTTAC | 1743 | + | part of a light responsive element |
| *BoESP3*^cabbage^ | circadian | CAAAGATATC | 1802 | - | cis-acting regulatory element involved in circadian control |
| *BoESP3*^cabbage^ | ATC-motif | AGTAATCT | 1939 | - | part of a conserved DNA module involved in light responsiveness |
| *BoESP3*^cabbage^ | TCA-element | CCATCTTTTT | 1977 | - | cis-acting element involved in salicylic acid responsiveness |
| *BoESP4*^cabbage^ | P-box | CCTTTTG | 41 | + | gibberellin-responsive element |
| *BoESP4*^cabbage^ | LTR | CCGAAA | 57 | + | cis-acting element involved in low-temperature responsiveness |
| *BoESP4*^cabbage^ | O2-site | GATGACATGG | 75 | + | cis-acting regulatory element involved in zein metabolism regulation |
| *BoESP4*^cabbage^ | AuxRR-core | GGTCCAT | 81 | - | cis-acting regulatory element involved in auxin responsiveness |
| *BoESP4*^cabbage^ | CGTCA-motif | CGTCA | 170 | - | cis-acting regulatory element involved in the MeJA-responsiveness |
| *BoESP4*^cabbage^ | TGACG-motif | TGACG | 170 | + | cis-acting regulatory element involved in the MeJA-responsiveness |
| *BoESP4*^cabbage^ | CGTCA-motif | CGTCA | 191 | - | cis-acting regulatory element involved in the MeJA-responsiveness |
| *BoESP4*^cabbage^ | TGACG-motif | TGACG | 191 | + | cis-acting regulatory element involved in the MeJA-responsiveness |
| *BoESP4*^cabbage^ | CGTCA-motif | CGTCA | 212 | - | cis-acting regulatory element involved in the MeJA-responsiveness |
| *BoESP4*^cabbage^ | TGACG-motif | TGACG | 212 | + | cis-acting regulatory element involved in the MeJA-responsiveness |
| *BoESP4*^cabbage^ | ARE | AAACCA | 243 | - | cis-acting regulatory element essential for the anaerobic induction |
| *BoESP4*^cabbage^ | ARE | AAACCA | 313 | - | cis-acting regulatory element essential for the anaerobic induction |
| *BoESP4*^cabbage^ | ARE | AAACCA | 474 | - | cis-acting regulatory element essential for the anaerobic induction |
| *BoESP4*^cabbage^ | TCT-motif | TCTTAC | 666 | + | part of a light responsive element |
| *BoESP4*^cabbage^ | GT1-motif | GGTTAA | 931 | + | light responsive element |
| *BoESP4*^cabbage^ | ARE | AAACCA | 1030 | - | cis-acting regulatory element essential for the anaerobic induction |
| *BoESP4*^cabbage^ | ARE | AAACCA | 1036 | - | cis-acting regulatory element essential for the anaerobic induction |
| *BoESP4*^cabbage^ | TC-rich repeats | GTTTTCTTAC | 1062 | - | cis-acting element involved in defense and stress responsiveness |
| *BoESP4*^cabbage^ | ARE | AAACCA | 1123 | - | cis-acting regulatory element essential for the anaerobic induction |
| *BoESP4*^cabbage^ | ARE | AAACCA | 1129 | - | cis-acting regulatory element essential for the anaerobic induction |
| *BoESP4*^cabbage^ | ARE | AAACCA | 1202 | - | cis-acting regulatory element essential for the anaerobic induction |
| *BoESP4*^cabbage^ | TC-rich repeats | ATTCTCTAAC | 1329 | + | cis-acting element involved in defense and stress responsiveness |
| *BoESP4*^cabbage^ | G-box | TACGTG | 1375 | + | cis-acting regulatory element involved in light responsiveness |
| *BoESP4*^cabbage^ | ABRE | ACGTG | 1376 | + | cis-acting element involved in the abscisic acid responsiveness |
| *BoESP4*^cabbage^ | G-box | CCACGTAA | 1442 | - | cis-acting regulatory element involved in light responsiveness |
| *BoESP4*^cabbage^ | G-box | TACGTG | 1443 | + | cis-acting regulatory element involved in light responsiveness |
| *BoESP4*^cabbage^ | ABRE | ACGTG | 1444 | + | cis-acting element involved in the abscisic acid responsiveness |
| *BoESP4*^cabbage^ | MBSI | TTTTTACGGTTA | 1454 | - | MYB binding site involved in flavonoid biosynthetic genes regulation |
| *BoESP4*^cabbage^ | G-box | CACGAC | 1649 | + | cis-acting regulatory element involved in light responsiveness |
| *BoESP4*^cabbage^ | G-box | CACGAC | 1769 | - | cis-acting regulatory element involved in light responsiveness |
| *BoESP4*^cabbage^ | TC-rich repeats | GTTTTCTTAC | 1807 | + | cis-acting element involved in defense and stress responsiveness |
| *BoESP4*^cabbage^ | CGTCA-motif | CGTCA | 1877 | - | cis-acting regulatory element involved in the MeJA-responsiveness |
| *BoESP4*^cabbage^ | TGACG-motif | TGACG | 1877 | + | cis-acting regulatory element involved in the MeJA-responsiveness |
| *BoESP4*^cabbage^ | G-box | TAACACGTAG | 1936 | + | cis-acting regulatory element involved in light responsiveness |
| *BoESP4*^cabbage^ | Box II | ACACGTAGA | 1938 | + | part of a light responsive element |
| *BoESP4*^cabbage^ | ABRE | ACGTG | 1939 | - | cis-acting element involved in the abscisic acid responsiveness |
| *BoESP4*^cabbage^ | G-box | TACGTG | 1939 | - | cis-acting regulatory element involved in light responsiveness |

Supplementary Table 6 Effect of different hormone treatments on the content of glucosinolate breakdown products.

| Name | Data type | Mock | 200 μmol/L ABA | 50 μmol/L GA_3_ |
| --- | --- | --- | --- | --- |
| 2Prop-CN | mean | 0.1092 | 0.1946 | 0.1536 |
|  | SD | 0.0111 | 0.0108 | 0.0109 |
| 3But-CN | mean | 0.2037 | 0.5086 | 0.2674 |
|  | SD | 0.0326 | 0.1148 | 0.0091 |
| 2Prop-ITC | mean | 1.4637 | 0.1500 | 0.2787 |
|  | SD | 0.0539 | 0.1515 | 0.0676 |
| 3But-ITC | mean | 2.0741 | 0.3866 | 0.6191 |
|  | SD | 0.0225 | 0.1380 | 0.0601 |
| CETP | mean | 1.3706 | 3.5011 | 2.2444 |
|  | SD | 0.2107 | 0.1936 | 0.1455 |
| 3MTP-CN | mean | 0.1071 | 0.2516 | 0.1674 |
|  | SD | 0.0177 | 0.0041 | 0.0292 |
| CETB | mean | 4.4063 | 9.8750 | 6.6282 |
|  | SD | 0.7343 | 0.1262 | 1.6049 |
| 4MTB-CN | mean | 0.3241 | 0.9736 | 0.6195 |
|  | SD | 0.0590 | 0.0431 | 0.0658 |
| 3MTP-ITC | mean | 0.0865 | 0.1753 | 0.0496 |
|  | SD | 0.0013 | 0.0197 | 0.0074 |
| 4MTB-ITC | mean | 0.1035 | 0.3065 | 0.1265 |
|  | SD | 0.0138 | 0.2565 | 0.0685 |
| OZT | mean | 0.0701 | 0.1134 | 0.1165 |
|  | SD | 0.0084 | 0.0359 | 0.0059 |
| CHETB | mean | 0.2042 | 0.2054 | 0.1744 |
|  | SD | 0.0204 | 0.0372 | 0.0148 |
| 4MSOB-CN | mean | 0.1420 | 0.1504 | 0.1186 |
|  | SD | 0.0127 | 0.0021 | 0.0009 |
| 3MSOP-ITC | mean | 0.3495 | 1.0644 | 0.6399 |
|  | SD | 0.0531 | 0.1841 | 0.0831 |
| 4MSOB-ITC | mean | 0.3825 | 0.1270 | 0.1231 |
|  | SD | 0.0166 | 0.0635 | 0.0220 |

2Prop-CN: 3-butenyl nitrile; 3But-CN: 4-pentenenitrile; 2Prop-ITC: 2-propenyl ITC; 3But-ITC: 3-butenyl ITC; CETP: 1-cyano-2,3-epithiopropane; 3MTP-CN: 4-(methylthio)butanenitrile; CETB: 1-cyano-3,4-epithiobutane; 4MTB-CN: 5-(methylthio)pentanenitrile; 3MTP-ITC: 3-(methylthio)propyl ITC; 4MTB-ITC: 4-(methylthio)butyl ITC; OZT: 5-vinyl-1,3-oxazolidine-2-thione; CHETB: 3-hydroxy-4,5-epithiopentane; 4MSOB-CN: 5-(methylsulfinyl)pentanenitrile; 3MSOP-ITC: 3-(methylsulphinyl)propyl ITC; 4MSOB-ITC: 4-(methylsulfinyl)butyl ITC.
